# Paracetamol exposure during pregnancy, the risk of major congenital malformations, and perinatal and postnatal outcomes: a population-based cohort study

**DOI:** 10.1093/hropen/hoag037

**Published:** 2026-04-29

**Authors:** Daphna Idan, Ariel Avraham Hasidim, Itamar Ben Shitrit, Tal Michael, Amalia Levy, Gali Pariente, Eitan Lunenfeld, Sharon Daniel

**Affiliations:** Department of Epidemiology, Biostatistics, and Community Health Sciences, School of Public Health, Faculty of Health Sciences, Ben-Gurion University of the Negev, Beer-Sheva, Israel; Department of Epidemiology, Biostatistics, and Community Health Sciences, School of Public Health, Faculty of Health Sciences, Ben-Gurion University of the Negev, Beer-Sheva, Israel; Department of Pediatrics A, Schneider Children’s Medical Center of Israel, Petah Tikva, Israel; Gray Faculty of Medicine, Tel Aviv University, Tel Aviv, Israel; Department of Epidemiology, Biostatistics, and Community Health Sciences, School of Public Health, Faculty of Health Sciences, Ben-Gurion University of the Negev, Beer-Sheva, Israel; Clinical Research Center, Faculty of Health Sciences, Soroka University Medical Center, Ben-Gurion University of the Negev, Beer-Sheva, Israel; Department of Epidemiology, Biostatistics, and Community Health Sciences, School of Public Health, Faculty of Health Sciences, Ben-Gurion University of the Negev, Beer-Sheva, Israel; Department of Epidemiology, Biostatistics, and Community Health Sciences, School of Public Health, Faculty of Health Sciences, Ben-Gurion University of the Negev, Beer-Sheva, Israel; Department of Obstetrics and Gynecology, Faculty of Health Sciences, Ben-Gurion University of the Negev and Soroka University Medical Center, Beer-Sheva, Israel; Adelson School of Medicine, Ariel University, Ariel, Israel; Department of Epidemiology, Biostatistics, and Community Health Sciences, School of Public Health, Faculty of Health Sciences, Ben-Gurion University of the Negev, Beer-Sheva, Israel; Department of Pediatrics, Faculty of Health Sciences, Ben-Gurion University of the Negev, Beer-Sheva, Israel; Clalit Health Services, Southern District, Beer-Sheva, Israel

**Keywords:** paracetamol, acetaminophen, congenital malformations, birth defects, teratology, pharmacoepidemiology, drug safety, prenatal exposure, antipyretics, pain management

## Abstract

**STUDY QUESTION:**

Is maternal paracetamol exposure during the first and third trimesters of pregnancy associated with major congenital malformations and adverse perinatal and postnatal outcomes?

**SUMMARY ANSWER:**

Maternal paracetamol use during the first or third trimester was not associated with major congenital malformations or adverse perinatal and postnatal outcomes.

**WHAT IS KNOWN ALREADY:**

Paracetamol is the most commonly used analgesic and antipyretic during pregnancy and has long been considered safe. However, recent concerns have been raised regarding potential fetal and perinatal risks, with inconsistent findings across studies.

**STUDY DESIGN, SIZE, DURATION:**

In this population-based retrospective study, the cohort included 265 143 singleton pregnancies resulting in delivery or elective termination at a single tertiary medical center between 1998 and 2018. Separate analytic cohorts comprised 264 858 pregnancies for first-trimester analyses and 257 285 pregnancies for third-trimester analyses. Follow-up extended through delivery and the first year of life for congenital malformation ascertainment. There was no loss to follow-up for primary outcomes due to complete registry linkage.

**PARTICIPANTS/MATERIALS, SETTING, METHODS:**

All singleton pregnancies among women aged 15–45 years, insured by a regional health maintenance organization and managed at a tertiary university medical center, were included. Pregnancies with chromosomal or genetic abnormalities, teratogenic drug exposure, or multiple gestations were excluded. Paracetamol exposure (prescription and over-the-counter dispensations) was assessed separately for the first trimester (≤13 weeks) and third trimester (≥27 weeks) and categorized by total defined daily doses. Exposed and unexposed pregnancies were compared using multivariable Poisson regression and propensity score-based generalized full matching. Matching incorporated maternal demographics, comorbidities, pregnancy characteristics, and indications for paracetamol use. After matching, covariate balance was achieved (standardized mean differences <0.1 for all variables).

**MAIN RESULTS AND THE ROLE OF CHANCE:**

During the first trimester, paracetamol exposure was recorded in 41 011 pregnancies (15.5%); major congenital malformations occurred in 7.9% of exposed versus 6.9% of unexposed pregnancies (crude RR 1.14; 95% CI, 1.1–1.18), with no association after matching (adjusted RR 1.04; 95% CI, 0.98–1.10) and no associations with organ-specific malformations. Third-trimester exposure was recorded in 36 375 pregnancies (14.1%) and was not associated, in the matched analyses, with preterm birth, low or very low birth weight, perinatal death, low Apgar scores, or markers of premature ductus arteriosus closure or neonatal renal impairment. Dose–response and sensitivity analyses showed no evidence of increased risk, and the results were robust to plausible levels of exposure misclassification.

**LIMITATIONS, REASONS FOR CAUTION:**

Paracetamol exposure was based on dispensation rather than confirmed intake, and miscarriages were not captured. Exposure misclassification due to unrecorded over-the-counter use is possible; however, sensitivity analyses suggest it is unlikely to materially affect the findings.

**WIDER IMPLICATIONS OF THE FINDINGS:**

These findings are consistent with large cohort studies and contribute to the evidence supporting the relative safety of paracetamol use during early and late pregnancy.

**STUDY FUNDING/COMPETING INTEREST(S):**

No funding was received for this study. All authors have no conflicts of interest to declare.

**TRIAL REGISTRATION NUMBER:**

N/A.

WHAT DOES THIS MEAN FOR PATIENTS? Paracetamol is one of the most used medications during pregnancy, often taken for pain or fever. Some recent studies have raised concerns about whether it might affect the developing baby. In this study, we looked at health records from over 250 000 pregnancies to find out whether women who took paracetamol during early or late pregnancy had a higher chance of having a baby with birth defects or other health problems at birth. We found that taking paracetamol during pregnancy was not linked to a higher risk of birth defects or complications for the baby. While no medication should be taken unnecessarily during pregnancy, these results are reassuring for women who need paracetamol for pain or fever relief during pregnancy.

## Introduction

Pain and fever are common concerns during pregnancy. Chronic and acute pain conditions affect up to 25% of pregnancies, with pregnancy-related pain occurring in 22–72% (Interrante *et al.*, 2017). Fetal safety concerns may discourage analgesic use during pregnancy (Twigg *et al.*, 2016), potentially resulting in undertreated pain and fever and increased maternal distress. Maternal fever during pregnancy has been reported to be associated with adverse perinatal outcomes, including neurodevelopmental abnormalities and congenital malformations. However, the underlying pathophysiological mechanisms linking fever and major congenital malformations (MCMs) remain incompletely understood (Oster *et al.*, 2011; Dreier *et al.*, 2014; Sass *et al.*, 2017; Black *et al.*, 2019; Antoun *et al.*, 2021; Hcini *et al.*, 2023).

Paracetamol is the preferred analgesic and antipyretic during pregnancy (Food and Drug Administration, 2015; European Medicines Agency, 2020), used by more than half of pregnant women worldwide and up to 65% in the USA (Werler *et al.*, 2005). While historically considered safe, this assumption has recently been challenged. A 2021 Nature Reviews Endocrinology consensus statement cited evidence suggesting possible associations with neurodevelopmental, reproductive, and urogenital disorders (Bauer *et al.*, 2021). Subsequently, in September 2025, regulatory advisories in the USA addressed potential adverse pregnancy outcomes associated with paracetamol use during gestation (The White House, 2025).

Evidence regarding paracetamol exposure and MCMs remains inconsistent. A Danish cohort of over 88 000 pregnancies found no association between first-trimester exposure and overall risk of malformations, except for a rare ear–face–neck abnormality (Feldkamp *et al.*, 2010). Similarly, a US case–control study of more than 7000 pregnancies reported no increased risk of neural tube defects, cleft lip/palate, and gastroschisis in women who used paracetamol to treat fever (Rebordosa *et al.*, 2008).

Studies of third-trimester exposure have also yielded inconsistent results. A Scottish retrospective cohort study of 151 141 pregnancies reported associations with preterm delivery, stillbirth, abnormal birth weight, and low Apgar scores (Zafeiri *et al.*, 2022). Similarly, a Korean cohort of 41 440 pregnancies found increased risks of stillbirth, low Apgar scores, and high birth weight (Xu *et al.*, 2024), and a Danish study reported increased risks of preeclampsia and thromboembolic events (Rebordosa *et al.*, 2010). Conversely, a prospective German cohort study found no evidence of fetal cardiovascular or renal toxicity, including early ductus arteriosus closure (Dathe *et al.*, 2019). In addition, some studies reported decreased risks of low birth weight (LBW) and small for gestational age (SGA) (Castro *et al.*, 2022; De Castro *et al.*, 2022), whereas a case series identified a probable causal relationship with ductus arteriosus closure (Allegaert *et al.*, 2019).

This population-based cohort study examined the association between maternal paracetamol exposure during the first and third trimesters and the risk of MCMs, late pregnancy adverse outcomes, and neonatal outcomes, including early closure of the ductus arteriosus and renal insufficiency.

## Materials and methods

### Design, setting, and participants

This population-based cohort study was conducted within siPREG (Southern Israeli Pregnancy Registry), which tracks maternal and perinatal outcomes in southern Israel. The study adhered to STROBE guidelines (Vandenbroucke *et al.*, 2007) and received ethics approval from the Soroka University Medical Center (SUMC) committee in accordance with the Declaration of Helsinki (approval 0069-20-SOR; 7 March 2022).

We included all pregnancies among women aged 15–45 years insured by Clalit Health Services (CHS) in southern Israel that resulted in delivery (live or stillbirth) or elective pregnancy termination for suspected fetal malformations at SUMC between 1998 and 2018. The inclusion of pregnancies terminated due to suspected fetal malformations allows for the representation of cases otherwise excluded from live-birth cohorts, thereby preventing bias toward the null hypothesis (Levy *et al.*, 2012). Clalit covers ∼70% of women of reproductive age in the region, and SUMC accounts for nearly all deliveries (∼98%) in the district (Angel, 2007).

We excluded multiple gestations, pregnancies with known teratogenic drug exposures (isotretinoin, antimetabolites, and anti-epileptic drugs), and pregnancies with genetic or chromosomal anomalies.

### Exposure

Paracetamol exposure was defined as any dispensation, including both prescription and over-the-counter (OTC) purchases. Exposure was assessed separately for the first trimester (from the first day of the last menstrual period through the end of the 13th gestational week), the second trimester (gestational weeks 14–26), and the third trimester (after 27 weeks of gestation). For each period, exposure was quantified by the total number of defined daily doses (DDD) dispensed and categorized as no exposure (0 DDD), short-term (1–7 DDD), medium-term (8–21 DDD), and long-term (>21 DDD). The standard DDD for paracetamol was 3 g/day (WHO Collaborating Centre for Drug Statistics Methodology, 2024).

In Israel, most OTC purchases, including paracetamol, occur through health service clinics or pharmacies affiliated with state-mandated health maintenance organization (HMO), in this study, CHS. In these settings, medications are dispensed at reduced prices upon presentation of an HMO identification number, and all dispensations, whether prescription or OTC, are routinely captured in the computerized database.

### Outcomes

MCMs were identified based on the Metropolitan Atlanta Congenital Defects Program (MACDP) criteria and coded using the International Classification of Diseases, 9th Revision (ICD-9). We assessed the relationship between first-trimester paracetamol exposure and the overall occurrence of MCMs, as well as specific organ system categories, including cardiovascular (ICD-9: 745–747), central nervous system (740–743), musculoskeletal (754–756), gastrointestinal (750–751), and genitourinary anomalies (752–753).

We also evaluated third-trimester paracetamol exposure and its association with the following adverse outcomes: (i) perinatal outcomes including LBW (<2500 g), very-LBW (VLBW, <1500 g), low Apgar scores at 1 and 5 min (<7), preterm birth (<37 weeks), and perinatal death (antepartum, intrapartum, and postpartum); and (ii) surrogate markers for premature ductus arteriosus closure: patent ductus arteriosus (PDA, ICD-9 code 747.0), persistent fetal circulation (PFC, ICD-9 code 747.83), primary pulmonary hypertension (PPHN, ICD-9 code 416.0), and heart failure (HF, ICD-9 codes 428.x) (PDA was examined as a possible paradoxical outcome of intrauterine ductus arteriosus constriction; Dathe *et al.*, 2019); and (iii) markers of impaired renal functions: oligohydramnios (ICD-9 code 658.0) and acute kidney injury (AKI) in the first 30 days of life (ICD-9 codes 584.x and 586.x).

### Covariates

Paracetamol indications were identified using ICD-9 diagnostic codes recorded during the first trimester of pregnancy, corresponding to clinical conditions for which paracetamol is commonly used as an analgesic or antipyretic. These included febrile or infectious illnesses (e.g. general fever 780.6; respiratory and ENT infections 460–466, 381*, 462–463; urinary tract infections 599.0, 590*; other infectious or inflammatory conditions 041*, 079*, 682*, 614*, 615*), as well as pain-related conditions (e.g. headache 346, abdominal or pelvic pain 789, 623–625), musculoskeletal and joint disorders (e.g. back pain, myalgia, arthritis 714–716, 718–719, 724, 729), injuries or fractures (800–829, 840–848, 920–924), and pregnancy-related diagnoses potentially associated with analgesic use, such as threatened miscarriage (632). Women could have more than one indication, and all relevant first-trimester diagnoses were combined into a single composite indicator for paracetamol indication (yes/no).

Maternal obesity was defined as a binary variable based on the presence of any ICD-9 diagnosis in the 278.0–278.4 range recorded during pregnancy-related hospital encounters. Pre-gestational diabetes mellitus was identified using ICD-9 code 250*, and gestational diabetes mellitus (GDM) was identified using ICD-9 codes 648.00–648.04; both were modeled as categorical variables (yes/no). Folic acid use was defined as any dispensation during the first trimester and treated as a binary exposure. Maternal smoking status was classified as present or absent based on either self-reported smoking or documentation using ICD-9 code 305.1.

### Dataset assembly

The cohort was assembled by combining data from four different sources. Pregnancy and delivery information came from the SUMC Obstetrics and Gynaecology Division. MCM diagnoses within the first year of life were obtained from the SUMC hospitalization database. Malformations identified before elective terminations were manually collected from the SUMC Committee for Termination of Pregnancies registry. All diagnoses were verified by board-certified specialists and coded according to ICD-9. Medication dispensation data, including prescriptions and OTC paracetamol with Anatomical Therapeutic Chemical (ATC) codes and DDD, were retrieved from the CHS database. The databases were linked using national IDs and hospitalization numbers, to connect maternal, neonatal, and termination records.

### Statistical analyses

Categorical variables were presented as counts and percentages and compared between exposure groups using χ^2^ or Fisher’s exact tests, as appropriate. Continuous variables were summarized as means ± standard deviations or medians [IQR], depending on their distribution, and compared using two-sample *t*-tests or Wilcoxon rank-sum tests.

Adjusted, non-matched risk ratios (RRs) were estimated using multivariable Poisson regression models with a log link function. Marginal (average) effects were computed using G-computation, and robust variance estimates were obtained with cluster-robust heteroskedasticity-consistent (‘sandwich’) standard errors clustered by maternal identifier (Sun *et al.*, 1985; Zeileis, 2004, 2006; Zeileis *et al.*, 2020). All analyses were performed using the *marginaleffects* package (Arel-Bundock *et al.*, 2024).

For matched-adjusted analyses, we applied generalized full matching (Ho *et al.*, 2011; Sävje *et al.*, 2021) on the propensity score to further reduce confounding and estimate the average treatment effect (ATE) (Ho *et al.*, 2011). Propensity scores were calculated using a probit regression model including all covariates, achieving satisfactory covariate balance. Matching weights derived from this procedure were incorporated into the outcome model, with no units excluded during matching. Adjusted RRs were then obtained using G-computation based on weighted Poisson regression with two-way cluster robust variance estimation, employing cluster-robust standard errors by both matching subclasses and maternal identifier.

Variables incorporated into the models were predetermined by two experts (S.D. and A.A.H.) based on a directed acyclic graph representing the hypothesized relationships among the study variables (see Supplementary Figs S1, S2, and S3). Adjusted models for MCMs included maternal age, ethnicity, lack of perinatal care, diabetes, obesity, folic acid use, gravidity, maternal comorbidity indicating paracetamol treatment, and year of birth or termination. Models for perinatal outcomes were adjusted for maternal age, smoking, ethnicity, gravidity, lack of perinatal care, gestational age, infant sex, MCMs, diabetes, and year of birth or termination. Premature ductus arteriosus closure and renal impairment models were adjusted for infant sex, ethnicity, gestational age, maternal comorbidity indicating paracetamol treatment, and year of birth or termination.

### Bias and sensitivity analyses

A potential limitation of this study concerns the OTC availability of paracetamol. Purchases made at pharmacies not included in our data sources could result in some truly exposed women being incorrectly classified as unexposed, potentially biasing the estimates toward the null. However, in Israel, the majority of paracetamol sales occur through health service clinics or pharmacies affiliated with Clalit Health Services, and both prescription and non-prescription dispensations from these outlets are captured in our databases. Only a small number of independent pharmacies operate in the southern district, typically with higher retail prices, suggesting that missing exposure data are likely minimal. In a previous validation study conducted in the same source population (Daniel *et al.*, 2014), we estimated that relying on dispensation data may miss ∼1.3% of true paracetamol exposures.

To assess the potential influence of exposure misclassification due to unrecorded OTC paracetamol use, we performed a probabilistic sensitivity analysis (tipping-point). Under the assumption that dispensation records provide high specificity but incomplete sensitivity for paracetamol exposure, we treated documented exposure as accurate and simulated increasing levels of missed exposure by randomly reassigning individuals from the unexposed group to the exposed group. To model a conservative scenario in which misclassification could shift estimates away from the null, reclassified individuals were assigned the same prevalence of major malformations observed among recorded paracetamol-exposed pregnancies (7.9%). The proportion of reclassified individuals ranged from 0% to 3% of the total cohort in increments of 0.05%; for each increment, 100 random reallocations were conducted, and the primary matching-adjusted model was re-estimated to generate a distribution of effect estimates.

In addition, we evaluated dose–response using categorized DDD analysis. We also evaluated first- and second-trimester paracetamol exposure in relation to perinatal and neonatal adverse outcomes to explore potential differences in risk by exposure timing during pregnancy. Furthermore, we examined the distribution of paracetamol indication subgroups among exposed and unexposed pregnancies.

All statistical analyses were performed using R version 4.5.1 (R Core Team, 2025).

## Results

**Figure 1.**
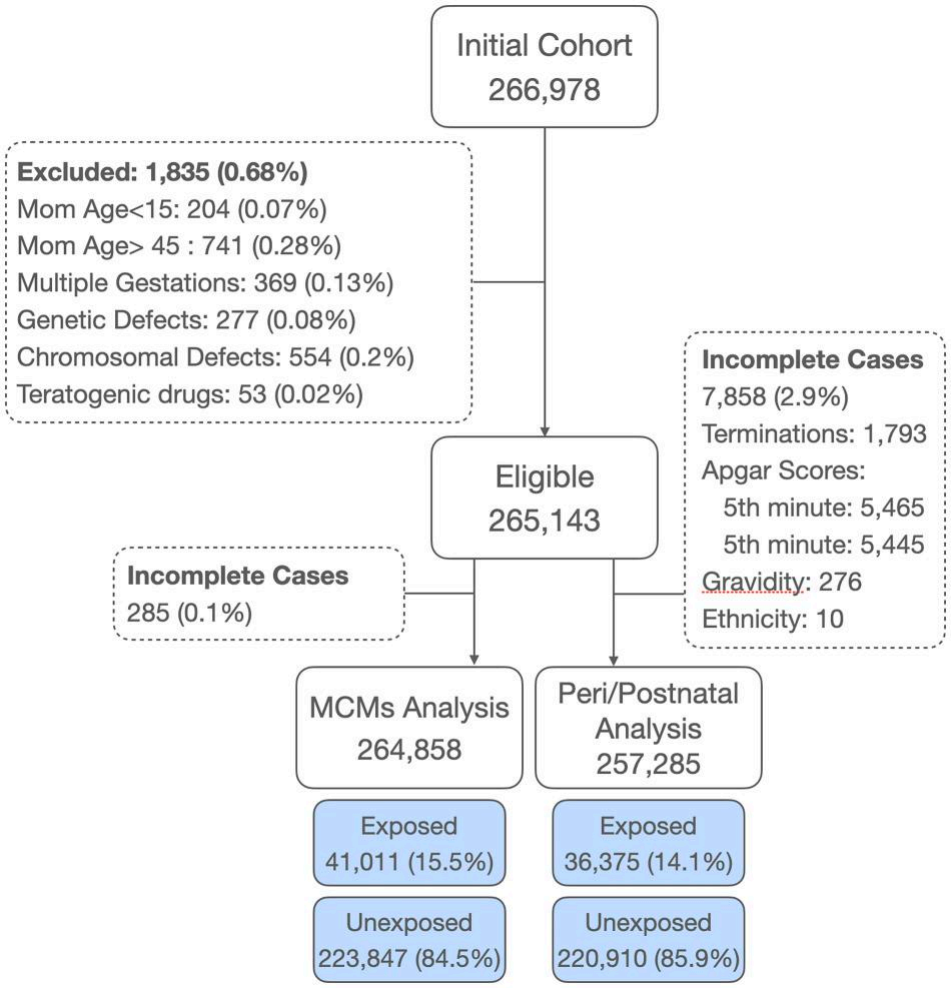
**Cohort selection flow diagram for first- and third-trimester paracetamol exposure analyses.** From an initial cohort of 266 978 pregnancies, 1835 (0.68%) were excluded due to maternal age outside 15–45 years, multiple gestations, genetic or chromosomal defects, or teratogenic drug exposure, leaving 265 143 pregnancies for inclusion. For the major congenital malformations (MCMs) analysis, 285 pregnancies (0.1%) with incomplete data were excluded, yielding an analytic cohort of 264 858 pregnancies, of which 41 011 (15.5%) were exposed to paracetamol during the first trimester and 223 847 (84.5%) were unexposed. For the peri/postnatal analysis, 7858 pregnancies (2.9%) with incomplete data (including terminations and missing Apgar scores, gravidity, or ethnicity data) were excluded, yielding an analytic cohort of 257 285 pregnancies, of which 36 375 (14.1%) were exposed to paracetamol during the third trimester and 220 910 (85.9%) were unexposed. MCMs, major congenital malformations.

**Figure 2.**
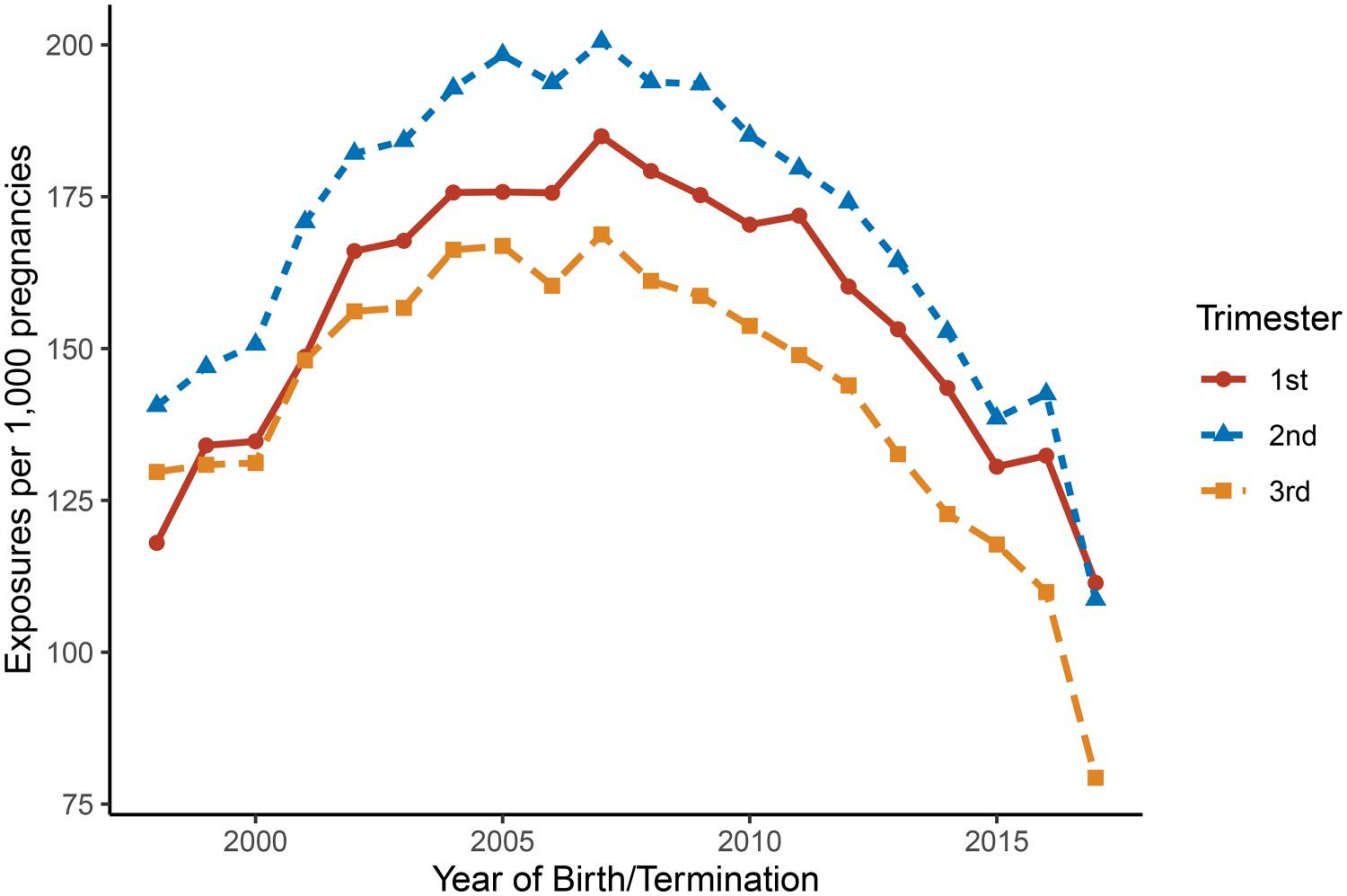
**Trends in paracetamol dispensation during pregnancy by trimester, 1998–2018.** Paracetamol exposure rates (per 1000 pregnancies) are shown separately for the first (solid line, circles), second (dashed line, triangles), and third (dashed line, squares) trimesters across the study period. Exposure rates across all three trimesters followed broadly parallel trends, rising from 1998 to ∼2005–2007 and declining thereafter through 2018. The second trimester consistently showed the highest exposure rates, followed by the first and third trimesters.

A total of 266 978 births and elective terminations occurred at SUMC between 1998 and 2018. Of these, 265 143 pregnancies met eligibility criteria (Fig. 1). Paracetamol exposure during pregnancy showed broadly parallel trends in the first and third trimesters (Fig. 2). First-trimester use increased until 2007, peaking at 185.0 exposures per 1000 pregnancies, then declined to 111.4 per 1000 by 2018. Third-trimester use followed a similar trajectory, peaking slightly lower at 168.8 exposures per 1000 pregnancies around 2005–2007, and decreasing to 79.3 per 1000 by 2018.

### First-trimester exposure and MCMs

Descriptive characteristics of the eligible study population, comprising 265 143 pregnancies with and without first-trimester exposure, are presented in Supplementary Table S1. In total, 264 858 complete cases were included in the analysis. Among those, 41 011 (15.5%) pregnancies were exposed to paracetamol during the first trimester, compared with 223 847 (84.5%) unexposed. Table 1 presents a comparison of pregnancy and maternal characteristics between pregnancies exposed and unexposed to paracetamol during the first trimester (all well-balanced after matching; absolute standardized mean difference [aSMD] <0.1). Before matching, exposed pregnancies were more often of Bedouin ethnicity (80% vs 51%, aSMD = 0.65), and had higher gravidity (median 4 vs 3, aSMD = 0.33), maternal obesity (1.0% vs 0.4%, aSMD = 0.07), and folic acid use (42% vs 12%, aSMD = 0.72). Pregnancies with comorbidities indicating paracetamol treatment were more common among exposed pregnancies (14.0% vs 8.9%, aSMD = 0.15).

**Table 1.** Maternal and pregnancy characteristics by first-trimester paracetamol exposure in the matched cohort for the congenital malformations analysis.

Characteristic	Paracetamol	Unexposed	aSMD before	aSMD after
	N = 41 011^1^	N = 223 847^1^		
**Calendar year of birth** *			0.03	0.07
1998–2002	8349 (20%)	50 973 (23%)		
2003–2007	10 918 (27%)	51 074 (23%)		
2008–2012	11 505 (28%)	55 687 (25%)		
2013–2017	10 239 (25%)	66 113 (30%)		
**Maternal delivery age, years** *			0.03	0.02
<20	877 (2.1%)	4871 (2.2%)		
20–24	8753 (21%)	45 581 (20%)		
25–29	12 861 (31%)	69 165 (31%)		
30–34	10 585 (26%)	59 049 (26%)		
35–39	6090 (15%)	34 055 (15%)		
40–44	1783 (4.3%)	10 668 (4.8%)		
≥45	62 (0.2%)	458 (0.2%)		
**Maternal ethnic group (Bedouin)** *	32 871 (80%)	113 343 (51%)	0.65	0.03
**Maternal obesity** *	418 (1.0%)	939 (0.4%)	0.07	0.02
**Maternal smoking during pregnancy**	174 (0.4%)	857 (0.4%)	0.01	0.02
**Maternal diabetes** *	317 (0.8%)	676 (0.3%)	0.06	0.01
**Maternal comorbidity indicating Paracetamol Tx** *	5596 (14.0%)	19 984 (8.9%)	0.15	0.02
**Gravidity** *	4 (2, 6)	3 (2, 5)	0.33	0.02
**Gestational age, weeks**	39.4 (38.0, 40.3)	39.3 (38.0, 40.1)	0.07	0.05
Missing	100	611		
**Conception by assisted reproductive technology**	169 (0.4%)	750 (0.3%)	0.01	0.01
Conception by insemination	39 (<0.1%)	146 (<0.1%)	0.01	0
Conception by IVF	145 (0.4%)	648 (0.3%)	0.01	0.01
**Lack of prenatal care** *	420 (1.0%)	1401 (0.6%)	0.04	0
**Folic acid** *	17 088 (42%)	26 204 (12%)	0.72	0.03
**Sex of newborn (males)**	20 807 (51%)	114 116 (51%)	0.01	0
Missing	73	1555		
**Pregnancy termination**	73 (0.2%)	1555 (0.7%)	0.08	0.05

1 n (%); Median (Q1, Q3).

* Covariates included in adjusted models (The model was matched and adjusted for maternal age, ethnicity, lack of perinatal care, diabetes, obesity, folic acid supplementation, gravidity, calendar year, and maternal paracetamol indication).

aSMD, absolute standardized mean difference; ATE, average treatment effect; ESS, effective sample size; Tx, treatment.

ESS for matching-adjusted model targeting the ATE were 14 600 exposed and 202 645 controls. No actual units were discarded during the process.

**Figure 3.**
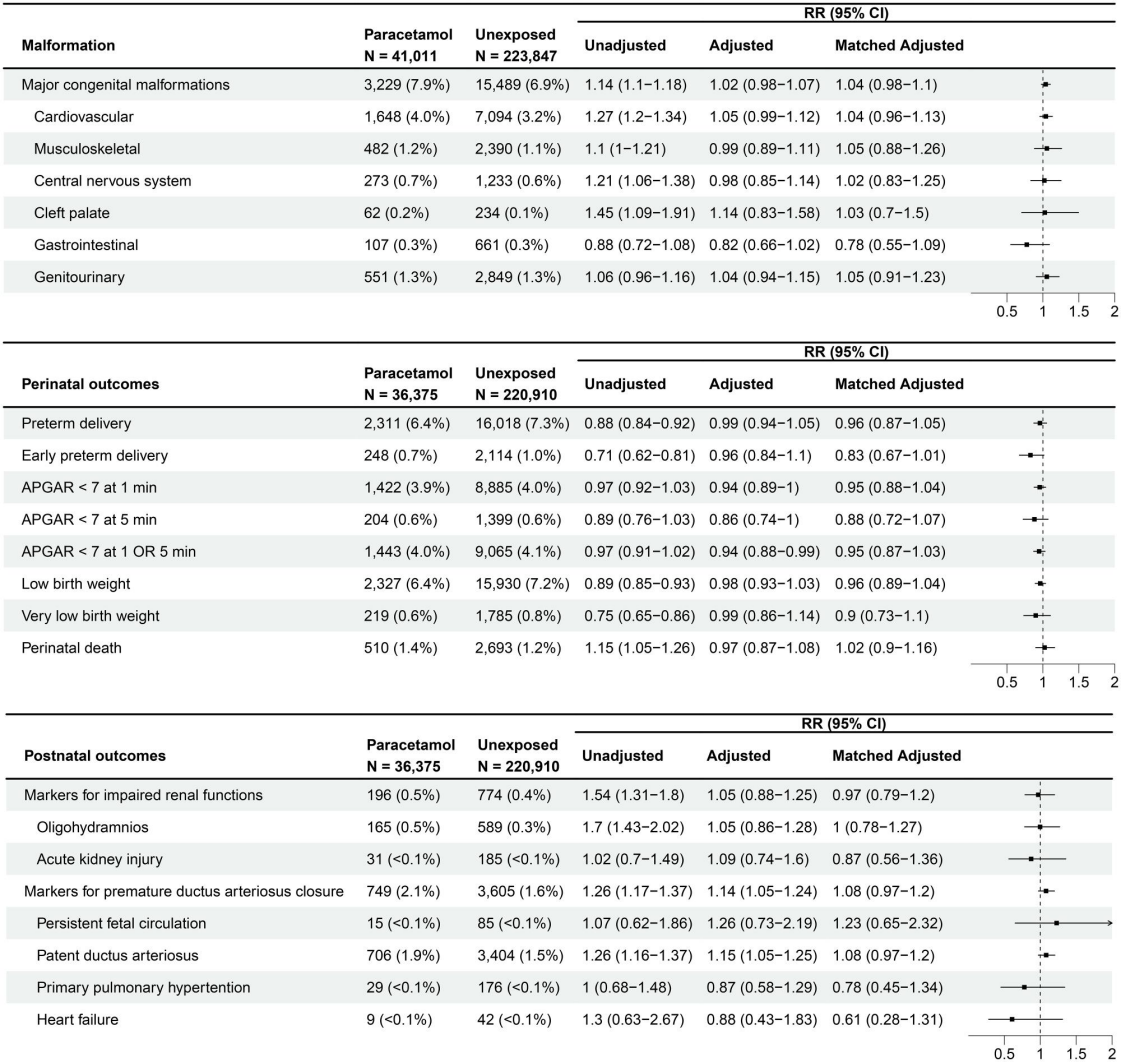
**Associations between first-trimester paracetamol exposure and major congenital malformations and between third-trimester exposure and adverse perinatal and postnatal outcomes.** RRs with 95% confidence intervals (CIs) are presented for unadjusted, multivariable-adjusted, and propensity score matched-adjusted analyses. The top panel shows associations between first-trimester paracetamol exposure (N = 41 011 exposed; N = 223 847 unexposed) and major congenital malformations, including overall and organ-specific categories. The middle panel shows associations between third-trimester exposure (N = 36 375 exposed; N = 220 910 unexposed) and perinatal outcomes, including preterm delivery, low Apgar scores, low birth weight, and perinatal death. The bottom panel shows associations between third-trimester exposure and postnatal outcomes, including markers for impaired renal function and markers for premature ductus arteriosus closure. Solid squares represent point estimates, with horizontal lines indicating 95% CIs. The vertical dashed line at RR = 1.0 represents the null. Models for malformations were adjusted for maternal age, ethnicity, lack of perinatal care, diabetes, obesity, folic acid use, gravidity, paracetamol indication, and year of birth or termination. Models for perinatal outcomes were adjusted for maternal age, smoking, ethnicity, gravidity, lack of perinatal care, gestational age, infant sex, major congenital malformations, diabetes, and year of birth or termination. Postnatal outcome models were adjusted for infant sex, ethnicity, gestational age, paracetamol indication, and year of birth or termination. RR, risk ratio; MCMs, major congenital malformations; APGAR, Appearance, Pulse, Grimace, Activity, Respiration score.

MCMs were more prevalent in paracetamol-exposed pregnancies compared to unexposed pregnancies (7.9% vs 6.9%, *P* < 0.001). Paracetamol exposure was associated with an increased risk of MCMs in crude analyses (RR 1.14; 95% CI, 1.1–1.18); however, no association was found in the adjusted or matched-adjusted analysis (matched aRR 1.04; 95% CI, 0.98–1.1) (Fig. 3). Cardiovascular malformations were more prevalent in the exposed group (4.0% vs 3.2%, *P* < 0.001), with a higher crude risk, which did not persist in the matched analysis (matched aRR 1.04; 95% CI, 0.96–1.13). Similarly, cleft palate showed a higher crude risk, while in the matched analysis, no association was found (matched aRR 1.03; 95% CI, 0.7–1.5). No associations were observed in either crude or matched analyses for musculoskeletal, central nervous system, gastrointestinal, and genitourinary malformations (Fig. 3).

### Third-trimester exposure and perinatal and postnatal adverse outcomes

Descriptive characteristics of the eligible study population, comprising 265 143 pregnancies with and without third-trimester exposure, are presented in Supplementary Table S2. In total, 257 185 complete cases were included in the perinatal and postnatal analyses. Among those, 36 375 (14.1%) pregnancies were exposed to paracetamol during the third trimester, compared with 220 910 (85.9%) unexposed. Table 2 presents a comparison of included exposed and unexposed pregnancies after matching for perinatal outcomes. Before matching, exposed pregnancies were more often of Bedouin ethnicity (80% vs 51%, aSMD = 0.64), had higher gravidity (median 4 vs 3, aSMD = 0.38), and were more likely to use folic acid (40% vs 13%, aSMD = 0.65). Pregnancies with diagnoses indicating paracetamol treatment were more common among exposed pregnancies (14% vs 9%, aSMD = 0.16). After matching for perinatal outcomes, all covariates in the model were well-balanced with aSMD < 0.1. Similarly, Supplementary Table S3 shows the same cohort but with balanced matching for postnatal outcomes.

**Table 2.** Maternal and pregnancy characteristics by third-trimester paracetamol exposure in the matched cohort for the perinatal outcomes analysis.

Characteristic	Paracetamol	Unexposed	aSMD before	aSMD after
	N = 36 375^1^	N = 220 910^1^		
Calendar year of birth*			0.1	0.04
1998–2002	8062 (22%)	49 519 (22%)		
2003–2007	9907 (27%)	50 082 (23%)		
2008–2012	10 059 (28%)	55 277 (25%)		
2013–2017	8347 (23%)	66 032 (30%)		
Maternal delivery age, years*			0.02	0.05
<20	724 (2.0%)	4883 (2.2%)		
20–24	7417 (20%)	45 528 (21%)		
25–29	11 259 (31%)	68 637 (31%)		
30–34	9500 (26%)	58 189 (26%)		
35–39	5690 (16%)	33 059 (15%)		
40–44	1720 (4.7%)	10 193 (4.6%)		
≥45	65 (0.2%)	421 (0.2%)		
Maternal ethnic group (Bedouin)*	29 048 (80%)	112 498 (51%)	0.64	0.02
**Maternal obesity**	367 (1.0%)	960 (0.4%)	0.07	0.05
Maternal smoking during pregnancy*	134 (0.4%)	789 (0.4%)	0	0.03
Maternal diabetes*	283 (0.8%)	647 (0.3%)	0.07	0.02
**Maternal comorbidity indicating Paracetamol Tx**	5054 (14%)	19 807 (9.0%)	0.16	0.11
Gravidity*	4 (2, 7)	3 (2, 5)	0.38	0.06
Gestational age, weeks*	39.6 (38.3, 40.4)	39.3 (38.0, 40.1)	0.08	0.01
**Conception by assisted reproductive technology**	99 (0.3%)	789 (0.4%)	0.02	0.01
Conception by insemination	21 (<0.1%)	161 (<0.1%)	0.01	0.01
Conception by IVF	80 (0.2%)	684 (0.3%)	0.02	0.01
Lack of prenatal care*	395 (1.1%)	1355 (0.6%)	0.05	0.02
**Folic acid**	14 389 (40%)	27 798 (13%)	0.65	0.69
Sex of newborn (males)*	18 491 (51%)	113 557 (51%)	0.01	0
Major congenital malformations*	2836 (7.8%)	15 037 (6.8%)	0.04	0.01

1 n (%); Median (Q1, Q3).

* Covariates included in adjusted models (The models were matched and adjusted for gestational age, sex of newborn, major congenital malformations, maternal age, ethnicity, lack of perinatal care, diabetes, smoking, gravidity, and calendar year).

aSMD, absolute standardized mean difference; ATE, average treatment effect; ESS, effective sample size; Tx, treatment.

ESS for matching-adjusted model targeting the ATE were 17 374 exposed and 212 399 controls. No actual units were discarded during the process.

Perinatal outcomes analysis showed no association between paracetamol exposure and risk of preterm or early preterm delivery, low or very low birth weight, or Apgar score <7 at 1 and 5 min, in either crude or matched analyses. Perinatal death was slightly more frequent among the exposed group (1.4% vs 1.2%) with an elevated crude risk, but the association was not observed after matching (aRR 1.02; 95% CI, 0.90–1.16, Fig. 3).

Furthermore, no significant association was found between paracetamol exposure and markers of impaired renal function (matched aRR 0.97; 95% CI, 0.79–1.2), with the rate of 0.5% vs 0.3% for oligohydramnios, and <0.1% of AKI for both groups. Exposure was also not associated with markers of premature ductus arteriosus closure (matched aRR 1.08; 95% CI, 0.97–1.2). PDA was most frequent (1.9% vs 1.5%), showing a crude but not matched association, while other markers, including PFC, PPHN, and HF each occurred in <0.1% and were not associated with paracetamol exposure (Fig. 3).

### Dose response analysis

The prevalence of MCMs among short-term (1–7 DDDs), medium-term (8–21 DDDs), and long-term (>21 DDDs) paracetamol users was 8.0%, 8.1%, and 10.2%, respectively, compared with 6.9% in the unexposed group (Table 3). No association with MCMs was found across dosage categories in adjusted analyses. Similarly, no association was found between third-trimester exposure as DDD categories and either perinatal or postnatal adverse outcomes (Supplementary Tables S4, S5, and S6).

**Table 3.** Risk of overall major congenital malformations by first-trimester paracetamol exposure categorized by defined daily dose (DDD).

Total DDD for paracetamol	Major congenital malformations	*Adjusted RR (95% CI)
None	15 905/230 405 (6.9%)	Reference
1–7	2141/26 828 (8.0%)	1.05 (1–1.1)
8–21	558/6898 (8.1%)	1.02 (0.94–1.11)
>21	45/441 (10.2%)	1.24 (0.89–1.6)

* Models were adjusted for maternal age, ethnicity, lack of perinatal care, diabetes, obesity, folic acid use, gravidity, maternal comorbidity indicating paracetamol treatment, and year of birth or pregnancy termination. Defined daily dose is a standardized measure representing the typical daily amount of a drug dispensed for use in adults. The standard defined daily dose for paracetamol was 3 g/day (WHO Collaborating Centre for Drug Statistics Methodology, 2024).

### Sensitivity analyses

To evaluate the possible influence of exposure misclassification, we performed simulations introducing varying degrees of misclassification, from 0.1% to 3% of pregnancies originally categorized as unexposed, with 100 bootstrap iterations at each level (with a 7.9% major malformation prevalence among reclassified individuals). Based on a previously identified 1.3% rate of undocumented OTC purchases, the estimated matched relative risk remained modest and non-significant (RR = 1.04, *P* = 0.08; 95% CI, 1.00–1.08), indicating that the effect of potential exposure misclassification on the study findings is minimal (Supplementary Fig. S4).

We did not detect associations between first and second trimester paracetamol exposure and perinatal and neonatal adverse outcomes (Supplementary Tables S7, S8, S9, S10, S11, and S12).

Regarding the distribution of indications among exposed and unexposed pregnancies, musculoskeletal and pain or inflammatory indications were rare in both groups (<0.1%). Injury or fracture-related indications were more frequent among exposed pregnancies (2.2% vs 1.4%). Pregnancy-related (10.0% vs 6.8%), and fever or infectious indications (3.5% vs 1.9%) were also more common among exposed compared with unexposed pregnancies (Supplementary Table S13).

## Discussion

In this population-based cohort including more than 265 000 singleton pregnancies, exposure to paracetamol during the first trimester was not associated with an increased risk of MCMs, either overall or by specific organ system. Consistently, no dose–response relationship was observed between cumulative paracetamol exposure and malformation risk. Third-trimester exposure was likewise not associated with preterm birth, low or very low birth weight, perinatal death, or early closure of the ductus arteriosus and kidney injury within the first 30 days of life. Across all analyses, findings remained stable after matching and adjustment for possible confounders and in sensitivity analyses addressing potential under ascertainment of OTC use.

Paracetamol use during the first and third trimesters peaked around 2005–2007 in an inverted U–shaped trend, likely reflecting evolving prescribing habits and growing awareness of potential risks or change in public health recommendations during pregnancy. The prevalence in our cohort was lower than some prior studies (15.5% first trimester, 14.1% third trimester), largely due to methodological differences. Many previous studies relied on maternal interviews or questionnaires, which yield higher estimates but are susceptible to recall bias (Werler *et al.*, 2005; Stergiakouli *et al.*, 2016; Bandoli *et al.*, 2020), whereas we used computerized healthcare data capturing recorded prescriptions and OTC dispensations, which typically report lower prevalence for commonly used OTC drugs. Additionally, our trimester-specific exposure windows limit direct comparability with studies defining exposure as ‘any use throughout pregnancy’ (Gram *et al.*, 2026). Finally, population-based estimates of ∼14% exposure during pregnancy have been reported, closely aligning with our findings (De Castro *et al.*, 2022).

Paracetamol exposure was higher among Bedouin women, consistent with prior reports of greater OTC medication use in this population (Kheiri *et al.*, 2020), and ethnicity may contribute to differences in both medication use and malformation prevalence through cultural practices, healthcare access, or prescribing patterns. Nonetheless, we matched and adjusted the models for ethnicity, thereby minimizing the likelihood that these differences influenced the observed associations. Obesity was defined using ICD-9 codes recorded during hospitalization, and the low proportion classified as obese likely reflects under-ascertainment, as obesity may not be coded when it is not the primary reason for admission; population-based surveys indicate ∼17% of adult Israeli women are obese (Keinan-Boker *et al.*, 2005), suggesting that our cohort likely underestimates the true prevalence of obesity among women of childbearing age.

Our findings align with several large population-based studies examining paracetamol safety in pregnancy. In a Danish National Birth Cohort (88 142 mother–child pairs), first-trimester exposure was not associated with overall malformations (Rebordosa *et al.*, 2008). The National Birth Defects Prevention Study, using maternal self-reports, (11 610 exposed cases) also found no excess risk of major defects, with protective associations observed for neural tube, craniofacial, and abdominal wall defects among febrile mothers (Feldkamp *et al.*, 2010). In contrast with these findings, a retrospective cohort study based on interview data suggested an association with gastroschisis, although concomitant medication use complicated interpretation (Werler *et al.*, 2002).

Regarding third-trimester outcomes, our null findings for preterm birth, low birth weight, and Apgar scores are consistent with other cohort studies demonstrating no adverse perinatal effects of paracetamol. The Ontario Birth Study, which also utilized self-reported exposure data, found no association between pregnancy paracetamol exposure and preterm birth, low birth weight or small-for-gestational-age outcomes (Arneja *et al.*, 2019). However, studies from China (n = 41 440) and Denmark (n = 63 833) reported increased risks including stillbirth, low Apgar scores, preeclampsia, and thromboembolic events (Rebordosa *et al.*, 2010; Xu *et al.*, 2024).

The question of early closure of the ductus arteriosus has generated considerable debate, particularly since paracetamol has increasingly been used as an alternative to ibuprofen for PDA closure in preterm infants, with some evidence suggesting comparable efficacy (Terrin *et al.*, 2016; Ohlsson and Shah, 2020). This has raised concerns regarding maternal paracetamol use during the third trimester; however, evidence linking such exposure to early ductal closure remains limited. A German observational study of 604 third-trimester paracetamol exposures found no clinically relevant risk of prenatal ductus arteriosus constriction or closure, nor evidence of fetal renal impairment (Dathe *et al.*, 2019), aligning with our findings. Conversely, a case-series review argued for a ‘likely’ causal link between late-pregnancy paracetamol and fetal ductal constriction based on 25 published cases (Allegaert *et al.*, 2019). Animal pharmacokinetic/pharmacodynamic modeling by Tanaka *et al.* suggested possible ductus constriction from third-trimester paracetamol exposure but indicated that actual risk may be minimal at low doses (Tanaka *et al.*, 2016). Our study, including 36 375 pregnancies exposed to paracetamol during the third trimester, found no evidence of premature ductus arteriosus closure.

By relying on dispensation data rather than retrospective self-report, our study minimizes recall bias and provides a more standardized assessment of paracetamol exposure during pregnancy. This methodological approach likely explains some of the variability in exposure prevalence and effect estimates reported across prior studies, rather than reflecting true inconsistency in findings. Heterogeneity in paracetamol use is substantial: for example, data from the MotherToBaby study revealed that 58% of users reported fewer than 10 days of use during pregnancy, while 9% reported 45 or more days, with considerable variation in indication and dose (Bandoli *et al.*, 2020). This heterogeneity emphasizes the importance of carefully characterizing exposure with respect to timing, dose, and duration when evaluating safety outcomes, as was done in the present analysis through dose-response assessment.

This study possesses several methodological strengths. The analysis encompassed a large, diverse population of over 265 000 pregnancies across a broad demographic spectrum. Inclusion of pregnancy terminations captured malformations that would be missed in birth-only cohorts, yielding more complete and less biased risk assessment (Levy *et al.*, 2012). Follow-up through the first year of life identified malformations diagnosed beyond the neonatal period, improving case ascertainment accuracy (Wik *et al.*, 2024). Dose–response analysis assessing cumulative exposure showed no trend toward increased malformation risk, supporting the robustness of primary findings. Matching for maternal, demographic, and pregnancy-related characteristics reduced the likelihood of residual confounding. Together, these methodological strengths enhance the validity and reliability of our results.

Several limitations warrant consideration. As with other pharmacoepidemiologic investigations, paracetamol exposure, including both prescription and OTC purchases, was ascertained from dispensation records rather than confirmed intake. While this approach captures recorded dispensations, it does not guarantee that the medication was actually taken, introducing potential misclassification of exposure. Prior research has demonstrated that, particularly among pregnant women, dispensation data have been validated as reliable sources for evaluating drug safety in pregnancy (Ray and Griffin, 1989; Johnson and Vollmer, 1991; West *et al.*, 1995; de Jonge *et al.*, 2015), including associations with congenital malformations (de Jong van den Berg *et al.*, 1999). However, we acknowledge that this validation has not been specifically established for paracetamol, and misclassification of exposure remains a potential limitation.

Second, although the study included malformations diagnosed in live births, stillbirths, and elective terminations, thereby covering the full spectrum of pregnancies with congenital malformations that came to medical attention, miscarriages were not captured in our dataset. This omission may lead to underestimation of early pregnancy losses potentially related to teratogenic exposures; however, miscarriage is inherently difficult to ascertain, particularly in early gestation when medical care may not be sought.

Third, OTC paracetamol availability could result in exposure misclassification. However, nearly all dispensations occur through Clalit or affiliated pharmacies, with only a small proportion of private pharmacies operating independently (Balak and Levi, 2024). Also, sensitivity analyses indicated that any resulting bias was minimal (see Materials and Methods). Although OTC paracetamol dispensations are recorded at the individual level, some purchases registered to pregnant women may have been intended for other household members, resulting in potential false-positive exposure classification. Such misclassification is expected to be non-differential with respect to study outcomes and would therefore tend to bias effect estimates toward the null. As a result, any true association between paracetamol exposure and adverse outcomes would likely be underestimated. The absence of a meaningful association in our analyses, despite this potential source of bias, supports the robustness of our findings.

Because indications for paracetamol use were identified from emergency department visits or hospitalizations rather than outpatient encounters, direct linkage between dispensations and their underlying indications was not possible, which may result in residual confounding. However, because no associations between paracetamol exposure and adverse outcomes were observed, the potential impact of residual confounding is likely limited.

In our data, we were unable to distinguish between prescription and OTC paracetamol use. These acquisition routes may differ with respect to actual treatment duration, dosing patterns, and clinical context; therefore, pregnancies classified as exposed likely include a mix of short-term or intermittent use and more sustained clinically indicated treatment. This heterogeneity in exposure intensity could attenuate true associations and bias effect estimates toward the null. In this context, the absence of an observed association in our analyses suggests that such misclassification is unlikely to have obscured a meaningful association.

## Conclusion

In this large population-based cohort, maternal paracetamol use was not associated with MCMs, adverse pregnancy outcomes, or early neonatal complications. These findings support the relative safety of paracetamol when used during pregnancy.

## Supplementary Material

hoag037_Supplementary_Data

## Data Availability

The data underlying this article cannot be shared publicly due to the presence of potentially identifiable health information and institutional privacy restrictions. The data were provided by Soroka University Medical Center under permission. De-identified data will be shared on reasonable request to the corresponding author, subject to approval by the Soroka University Medical Center Institutional Review Board.
