# Supplementary material for: Paracetamol exposure during pregnancy, the risk of major congenital malformations, and perinatal and postnatal outcomes: a population-based cohort study
Source: Hum Reprod Open. 2026 Apr 29;2026(3):hoag037. doi: 10.1093/hropen/hoag037 (PMC13195301; doi:10.1093/hropen/hoag037)
Supplement: hoag037_Supplementary_Data [file hoag037_supplementary_data.docx]

Supplementary Materials

**Paracetamol exposure during pregnancy, the risk of major congenital malformations, and perinatal and postnatal outcomes: a population-based cohort study**

Daphna Idan^1^†, Ariel Avraham Hasidim^1,2,3^†, Itamar Ben Shitrit^1,4^, Tal Michael^1^, Amalia Levy^1^, Gali Pariente^5^, Eitan Lunenfeld^6^, Sharon Daniel^1,7,8^*

^1^Department of Epidemiology, Biostatistics, and Community Health Sciences, School of Public Health, Faculty of Health Sciences, Ben-Gurion University of the Negev, Beer-Sheva, Israel
^2^Department of Pediatrics A, Schneider Children's Medical Center of Israel, Petah Tikva, Israel
^3^Gray Faculty of Medicine, Tel Aviv University, Tel Aviv, Israel
^4^Clinical Research Center, Faculty of Health Sciences, Soroka University Medical Center, Ben-Gurion University of the Negev, Beer-Sheva, Israel
^5^Department of Obstetrics and Gynecology, Faculty of Health Sciences, Ben-Gurion University of the Negev and Soroka University Medical Center, Beer-Sheva, Israel
^6^Adelson School of Medicine, Ariel University, Ariel, Israel
^7^Department of Pediatrics, Faculty of Health Sciences, Ben-Gurion University of the Negev, Beer-Sheva, Israel
^8^Clalit Health Services, Southern District, Beer-Sheva, Israel

† These authors contributed equally to this work.

*Corresponding Author:
Dr. Sharon Daniel
Department of Epidemiology, Biostatistics, and Community Health Sciences, School of Public Health, Faculty of Health Sciences, Ben-Gurion University of the Negev.
David Ben-Gurion Blvd 1, Beer-Sheva, 8410501, Israel
ORCID: [0000-0003-1820-9278](https://orcid.org/0000-0003-1820-9278)
Email: [daniels@post.bgu.ac.il](mailto:daniels@post.bgu.ac.il)

**Table of Contents**

[Supplementary Figure S1: Directed acyclic graph (DAG) describing the conceptual framework for major congenital malformations 3](#_Toc226909847)

[Supplementary Figure S2: Directed acyclic graph (DAG) describing the conceptual framework for adverse perinatal outcomes 4](#_Toc226909848)

[Supplementary Figure S3: Directed acyclic graph (DAG) describing the conceptual framework for adverse postnatal outcomes 5](#_Toc226909849)

[Supplementary Figure S4: Probabilistic sensitivity (tipping-point) analysis for exposure misclassification 10](#_Toc226909856)

[Supplementary Table S1. Baseline characteristics of first-trimester paracetamol-exposed and unexposed pregnancies population 6](#_Toc226909850)

[Supplementary Table S2. Baseline characteristics of third-trimester paracetamol-exposed and unexposed pregnancies population 7](#_Toc226909851)

[Supplementary Table S3. Maternal and pregnancy characteristics by third-trimester paracetamol exposure in the matched cohort for the postnatal outcomes analysis 8](#_Toc226909852)

[Supplementary Table S4. Risk of composite of adverse perinatal outcomes by third-trimester paracetamol exposure, categorized by defined daily dose (DDD) 9](#_Toc226909853)

[Supplementary Table S5. Risk of premature ductus arteriosus closure by third-trimester paracetamol exposure, categorized by defined daily dose (DDD) 9](#_Toc226909854)

[Supplementary Table S6. Risk of renal impairment closure by third-trimester paracetamol exposure, categorized by defined daily dose (DDD) 9](#_Toc226909855)

[Supplementary Table S7. Maternal and pregnancy characteristics by 1^st^ trimester paracetamol exposure in the matched cohort for the perinatal outcomes analysis 11](#_Toc226909857)

[Supplementary Table S8. Maternal and pregnancy characteristics by 1^st^ trimester paracetamol exposure in the matched cohort for the postnatal outcomes analysis 12](#_Toc226909858)

[Supplementary Table S9. Peri- and post-natal outcomes for 1^st^ trimester paracetamol exposure 13](#_Toc226909859)

[Supplementary Table S10. Maternal and pregnancy characteristics by 2^nd^ trimester paracetamol exposure in the matched cohort for the Perinatal outcomes analysis 14](#_Toc226909860)

[Supplementary Table S11. Maternal and pregnancy characteristics by 2^nd^ trimester paracetamol exposure in the matched cohort for the Postnatal outcomes analysis 15](#_Toc226909861)

[Supplementary Table S12. Peri- and post-natal outcomes for 2^nd^ trimester paracetamol exposure 16](#_Toc226909862)

[Supplementary Table S13. Distribution of Indications for Analgesic Use, Grouped by Category, in Exposed vs Unexposed Pregnancies 17](#_Toc226909863)

## Supplementary Figure S1: Directed acyclic graph (DAG) describing the conceptual framework for major congenital malformations


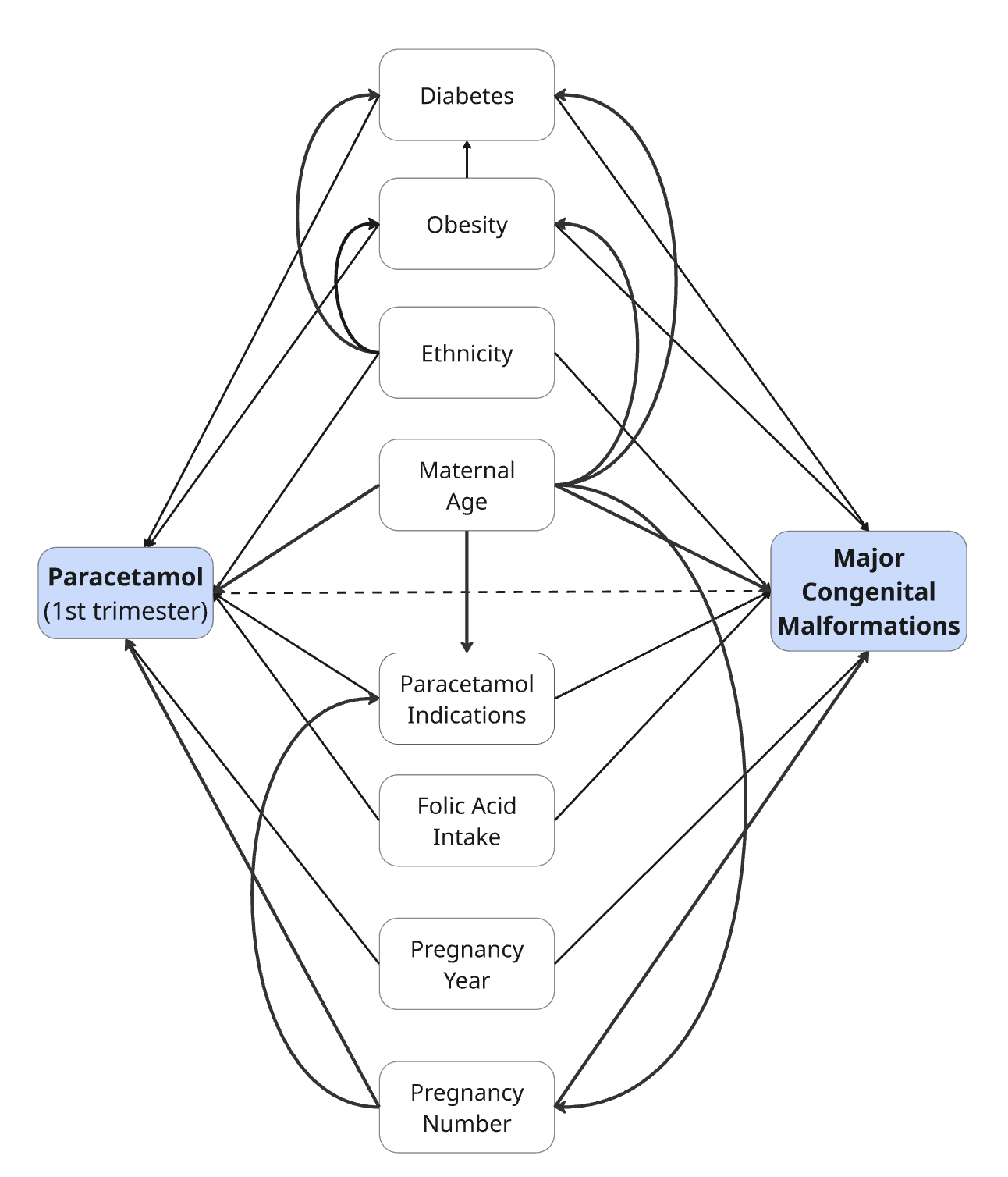


The DAG illustrates the assumed relationships between first-trimester paracetamol exposure and major congenital malformations. Solid arrows indicate hypothesized causal links, while the dashed arrow represents the primary association of interest.

## Supplementary Figure S2: Directed acyclic graph (DAG) describing the conceptual framework for adverse perinatal outcomes

**
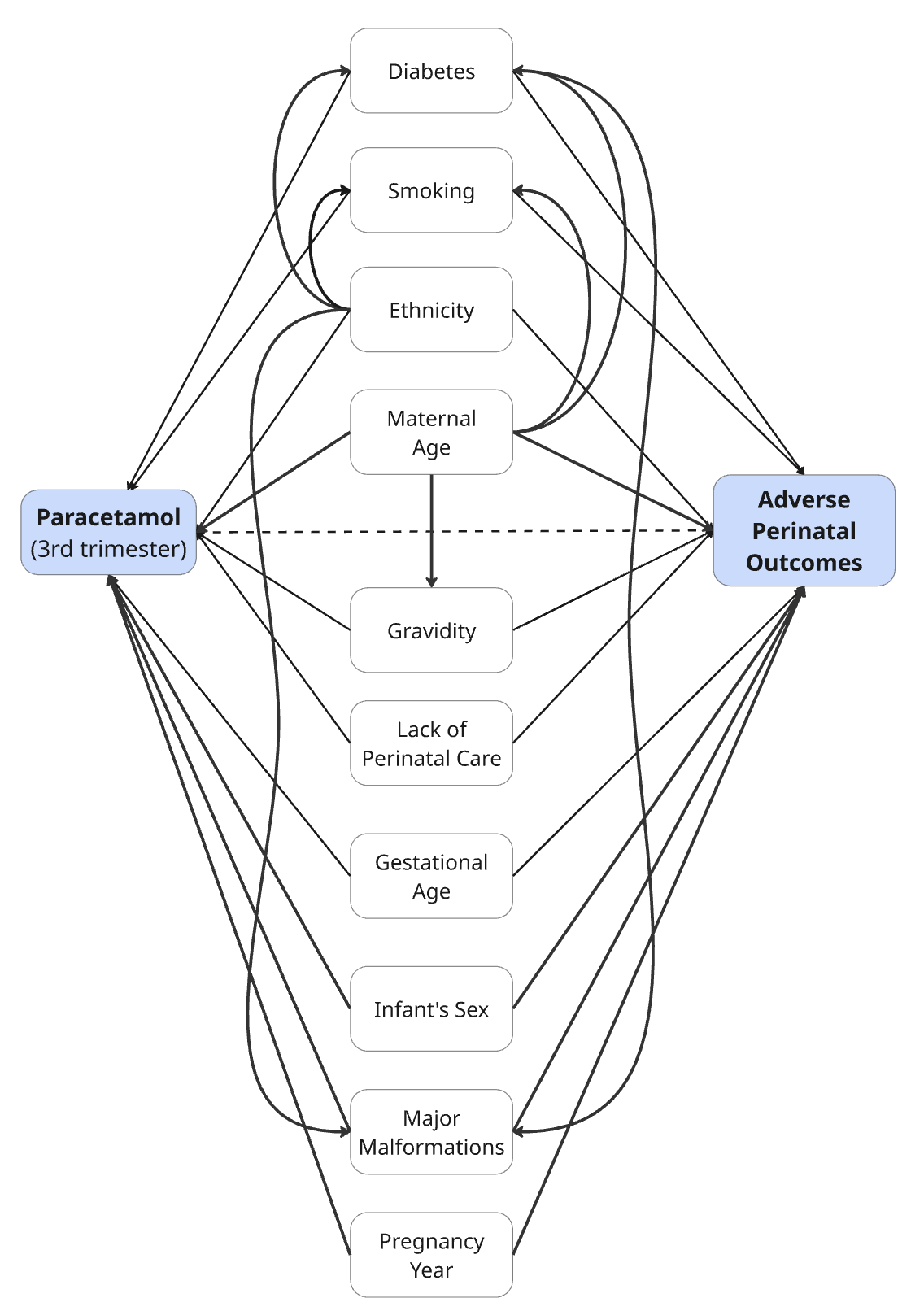
**

The DAG illustrates the assumed relationships between third-trimester paracetamol exposure and adverse perinatal outcomes. Solid arrows indicate hypothesized causal links, while the dashed arrow represents the primary association of interest.

## Supplementary Figure S3: Directed acyclic graph (DAG) describing the conceptual framework for adverse postnatal outcomes


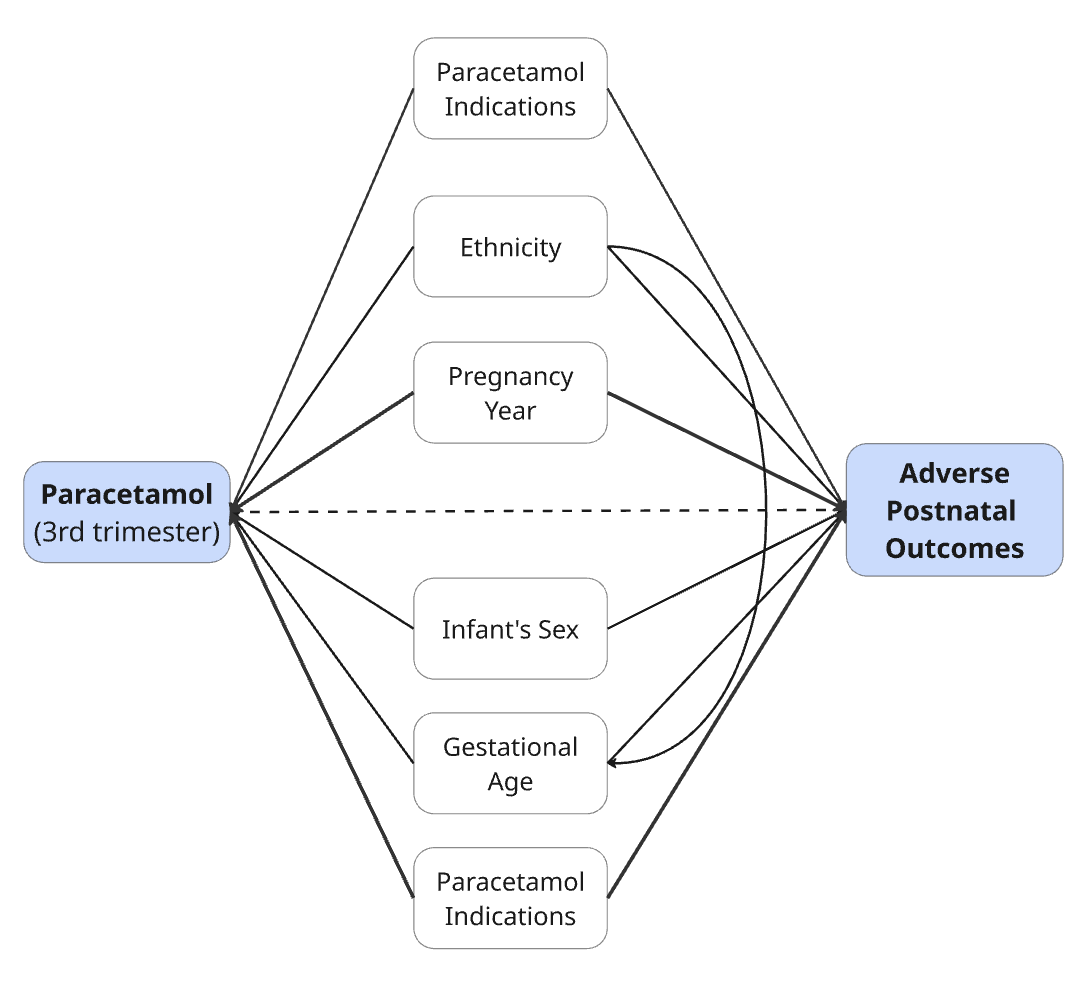


The DAG illustrates the assumed relationships between third-trimester paracetamol exposure and adverse postnatal outcomes. Solid arrows indicate hypothesized causal links, while the dashed arrow represent the primary association of interest.

## Supplementary Figure S4: Probabilistic sensitivity (tipping-point) analysis for exposure misclassification

Across simulations, the estimated risk increased progressively as larger proportions of the cohort were assumed to have unobserved paracetamol exposure (see figure below). Using a 1.3% missed-exposure rate derived from prior validation work at SUMC as a plausible scenario, reclassification of 1.3% of the cohort—assuming a 7.9% prevalence of major malformations among reassigned individuals—resulted in a modest and non-robust association in the matching-adjusted analysis (RR = 1.04, p = 0.08, 95%CI = 1.00–1.08).


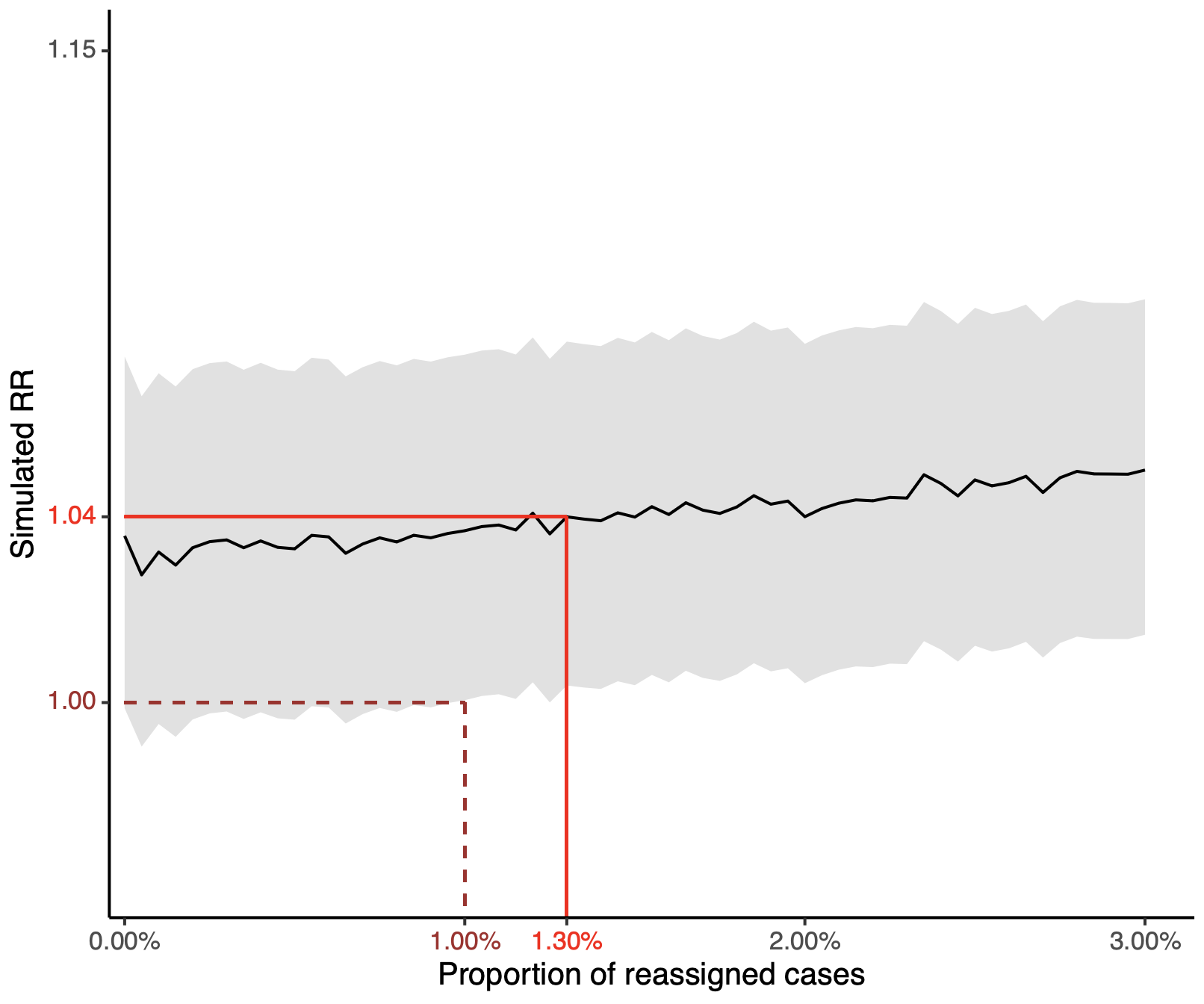


The figure presents the results of a probabilistic sensitivity analysis assessing the impact of potential misclassification of paracetamol exposure due to unrecorded over-the-counter use. Increasing proportions of individuals from the unexposed group were randomly reassigned to the exposed group (0–3% of the cohort), assuming a 7.9% prevalence of major congenital malformations among reassigned individuals. For each increment, 100 simulations were performed, and the matching-adjusted model was re-estimated. The analysis demonstrates the change in estimated risk and corresponding 95% confidence intervals as a function of increasing levels of assumed missed exposure.

## Supplementary Table S1. Baseline characteristics of first-trimester paracetamol-exposed and unexposed pregnancies population

| **Characteristic** | **Paracetamol** N = 41,032*^1^* | **Unexposed** N = 224,111*^1^* | **p-value***^2^* |
| --- | --- | --- | --- |
| **Calendar year of birth** |  |  | <0.001 |
| **1998–2002** | 8,352 (20%) | 51,025 (23%) |  |
| **2003–2007** | 10,922 (27%) | 51,179 (23%) |  |
| **2008–2012** | 11,510 (28%) | 55,768 (25%) |  |
| **2013–2017** | 10,248 (25%) | 66,139 (30%) |  |
| **Maternal delivery age, years** |  |  | <0.001 |
| **<20** | 877 (2.1%) | 4,896 (2.2%) |  |
| **20–24** | 8,755 (21%) | 45,666 (20%) |  |
| **25–29** | 12,867 (31%) | 69,232 (31%) |  |
| **30–34** | 10,591 (26%) | 59,104 (26%) |  |
| **35–39** | 6,094 (15%) | 34,080 (15%) |  |
| **40–44** | 1,785 (4.4%) | 10,675 (4.8%) |  |
| **≥45** | 63 (0.2%) | 458 (0.2%) |  |
| **Maternal ethnic group (Bedouin)** | 32,888 (80%) | 113,450 (51%) | <0.001 |
| Missing | 0 | 10 |  |
| **Maternal obesity** | 419 (1.0%) | 939 (0.4%) | <0.001 |
| **Maternal smoking during pregnancy** | 174 (0.4%) | 860 (0.4%) | 0.2 |
| **Maternal diabetes** | 317 (0.8%) | 677 (0.3%) | <0.001 |
| **Maternal comorbidity indicating Paracetamol Tx** | 5,601 (14%) | 19,992 (8.9%) | <0.001 |
| **Gravidity** | 4 (2, 6) | 3 (2, 5) | <0.001 |
| Missing | 21 | 255 |  |
| **Gestational age, weeks** | 39.4 (38.0, 40.3) | 39.3 (38.0, 40.0) | <0.001 |
| Missing | 104 | 643 |  |
| **Conception by assisted reproductive technology** | 169 (0.4%) | 750 (0.3%) | 0.014 |
| **Conception by insemination** | 39 (<0.1%) | 146 (<0.1%) | 0.035 |
| **Conception by IVF** | 145 (0.4%) | 648 (0.3%) | 0.028 |
| **Lack of prenatal care** | 421 (1.0%) | 1,402 (0.6%) | <0.001 |
| **Folic acid** | 17,100 (42%) | 26,209 (12%) | <0.001 |
| **Sex of newborn (males)** | 20,819 (51%) | 114,166 (51%) | 0.059 |
| Missing | 73 | 1,720 |  |
| **Pregnancy termination** | 73 (0.2%) | 1,720 (0.8%) | <0.001 |
| *^1^*n (%); Median (Q1, Q3) | | | |
| *^2^*Pearson's Chi-squared test; Wilcoxon rank sum test | | | |
| IVF, In Vitro Fertilization; Tx, Treatment. | | | |

## Supplementary Table S2. Baseline characteristics of third-trimester paracetamol-exposed and unexposed pregnancies population

| **Characteristic** | **Paracetamol** N = 37,333*^1^* | **Unexposed** N = 227,810*^1^* | **p-value***^2^* |
| --- | --- | --- | --- |
| **Calendar year of birth** |  |  | <0.001 |
| 1998–2002 | 8,277 (22%) | 51,100 (22%) |  |
| 2003–2007 | 10,167 (27%) | 51,934 (23%) |  |
| 2008–2012 | 10,293 (28%) | 56,985 (25%) |  |
| 2013–2017 | 8,596 (23%) | 67,791 (30%) |  |
| **Maternal delivery age, years** |  |  | 0.002 |
| <20 | 735 (2.0%) | 5,038 (2.2%) |  |
| 20–24 | 7,569 (20%) | 46,852 (21%) |  |
| 25–29 | 11,534 (31%) | 70,565 (31%) |  |
| 30–34 | 9,774 (26%) | 59,921 (26%) |  |
| 35–39 | 5,863 (16%) | 34,311 (15%) |  |
| 40–44 | 1,790 (4.8%) | 10,670 (4.7%) |  |
| ≥45 | 68 (0.2%) | 453 (0.2%) |  |
| **Maternal ethnic group (Bedouin)** | 29,868 (80%) | 116,470 (51%) | <0.001 |
| Missing | 0 | 10 |  |
| **Maternal obesity** | 378 (1.0%) | 980 (0.4%) | <0.001 |
| **Maternal smoking during pregnancy** | 142 (0.4%) | 892 (0.4%) | 0.7 |
| **Maternal diabetes** | 292 (0.8%) | 702 (0.3%) | <0.001 |
| **Maternal comorbidity indicating Paracetamol Tx** | 5,195 (14%) | 20,398 (9.0%) | <0.001 |
| **Gravidity** | 4 (2, 7) | 3 (2, 5) | <0.001 |
| Missing | 22 | 254 |  |
| **Gestational age, weeks** | 39.6 (38.3, 40.3) | 39.3 (38.0, 40.0) | <0.001 |
| Missing | 72 | 675 |  |
| **Conception by assisted reproductive technology** | 103 (0.3%) | 816 (0.4%) | 0.012 |
| **Conception by insemination** | 21 (<0.1%) | 164 (<0.1%) | 0.3 |
| **Conception by IVF** | 84 (0.2%) | 709 (0.3%) | 0.005 |
| **Lack of prenatal care** | 406 (1.1%) | 1,417 (0.6%) | <0.001 |
| **Folic acid** | 14,759 (40%) | 28,550 (13%) | <0.001 |
| **Sex of newborn (males)** | 18,914 (51%) | 116,071 (51%) | 0.035 |
| Missing | 65 | 1,728 |  |
| **Pregnancy termination** | 65 (0.2%) | 1,728 (0.8%) | <0.001 |
| *^1^*n (%); Median (Q1, Q3) | | | |
| *^2^*Pearson's Chi-squared test; Wilcoxon rank sum test | | | |
| IVF, In Vitro Fertilization; Tx, Treatment. | | | |

## Supplementary Table S3. Maternal and pregnancy characteristics by third-trimester paracetamol exposure in the matched cohort for the postnatal outcomes analysis

| **Characteristic** | **Paracetamol** N = 36,375*^1^* | **Unexposed** N = 220,910*^1^* | **aSMD before** | **aSMD after** |
| --- | --- | --- | --- | --- |
| **Calendar year of birth*^*^*** |  |  | 0.1 | 0.02 |
| 1998–2002 | 8,062 (22%) | 49,519 (22%) |  |  |
| 2003–2007 | 9,907 (27%) | 50,082 (23%) |  |  |
| 2008–2012 | 10,059 (28%) | 55,277 (25%) |  |  |
| 2013–2017 | 8,347 (23%) | 66,032 (30%) |  |  |
| **Maternal delivery age, years** |  |  | 0.02 | 0.15 |
| <20 | 724 (2.0%) | 4,883 (2.2%) |  |  |
| 20–24 | 7,417 (20%) | 45,528 (21%) |  |  |
| 25–29 | 11,259 (31%) | 68,637 (31%) |  |  |
| 30–34 | 9,500 (26%) | 58,189 (26%) |  |  |
| 35–39 | 5,690 (16%) | 33,059 (15%) |  |  |
| 40–44 | 1,720 (4.7%) | 10,193 (4.6%) |  |  |
| ≥45 | 65 (0.2%) | 421 (0.2%) |  |  |
| **Maternal ethnic group (Bedouin)*^*^*** | 29,048 (80%) | 112,498 (51%) | 0.64 | 0 |
| **Maternal obesity** | 367 (1.0%) | 960 (0.4%) | 0.07 | 0.03 |
| **Maternal smoking during pregnancy** | 134 (0.4%) | 789 (0.4%) | 0 | 0 |
| **Maternal diabetes** | 283 (0.8%) | 647 (0.3%) | 0.07 | 0.04 |
| **Maternal comorbidity indicating Paracetamol Tx*^*^*** | 5,054 (14%) | 19,807 (9.0%) | 0.16 | 0.01 |
| **Gravidity** | 4 (2, 7) | 3 (2, 5) | 0.38 | 0.22 |
| **Gestational age, weeks*^*^*** | 39.6 (38.3, 40.4) | 39.3 (38.0, 40.1) | 0.08 | 0.04 |
| **Conception by assisted reproductive technology** | 99 (0.3%) | 789 (0.4%) | 0.02 | 0.02 |
| **Conception by insemination** | 21 (<0.1%) | 161 (<0.1%) | 0.01 | 0.01 |
| **Conception by IVF** | 80 (0.2%) | 684 (0.3%) | 0.02 | 0.02 |
| **Lack of prenatal care** | 395 (1.1%) | 1,355 (0.6%) | 0.05 | 0 |
| **Folic acid** | 14,389 (40%) | 27,798 (13%) | 0.65 | 0.66 |
| **Sex of newborn (males)*^*^*** | 18,491 (51%) | 113,557 (51%) | 0.01 | 0 |
| *^1^*n (%); Median (Q1, Q3) | | | | |
| *^*^*Covariates included in adjusted models (The model was matched and adjusted for gestational age, sex of newborn, ethnicity, calendar year and maternal paracetamol indication). | | | | |
| aSMD, absolute Standardized Mean Difference; ATE, Average Treatment Effect; ESS, Effective Sample Size; IVF, In Vitro Fertilization; Tx, Treatment. | | | | |
| ESS for matching-adjusted model targeting the ATE were 18,108 exposed and 216,374 controls. No actual units were discarded during the proccess. | | | | |

## Supplementary Table S4. Risk of composite of adverse perinatal outcomes by third-trimester paracetamol exposure, categorized by defined daily dose (DDD)

| Total DDD for Paracetamol | Composite Negative Perinatal Outcomes^1^ | Adjusted RR (%95 CI) |
| --- | --- | --- |
| None | 32266/228031 (14.1%) | 1 (NA-NA) |
| 1-7 | 3173/23629 (13.4%) | 0.98 (0.95-1.02) |
| 8-21 | 734/5655 (13.0%) | 0.97 (0.9-1.03) |
| >21 | 40/393 (10.2%) | 0.93 (0.68-1.18) |

^1^Composite Negative Perinatal Outcomes: preterm delivery, early preterm delivery, APGAR < 7 at 1 min, APGAR < 7 at 1 or 5 min, low birth weight, very low birth weight, perinatal death, oligohydramnios, persistent fetal circulation, patent ductus arteriosus, primary pulmonary hypertension, heart failure.

## Supplementary Table S5. Risk of premature ductus arteriosus closure by third-trimester paracetamol exposure, categorized by defined daily dose (DDD)

| Total DDD for Paracetamol | Premature ductus arteriosus closure markers | Adjusted RR (%95 CI) |
| --- | --- | --- |
| None | 3829/234716 (1.6%) | Rerefence |
| 1-7 | 497/24244 (2.0%) | 1.12 (1.02-1.23) |
| 8-21 | 124/5778 (2.1%) | 1.15 (0.94-1.35) |
| >21 | 13/405 (3.2%) | 1.94 (0.9-2.97) |

## Supplementary Table S6. Risk of renal impairment closure by third-trimester paracetamol exposure, categorized by defined daily dose (DDD)

| Total DDD for Paracetamol | Renal impairment markers | Adjusted RR (%95 CI) |
| --- | --- | --- |
| None | 837/234716 (0.4%) | Rerefence |
| 1-7 | 140/24244 (0.6%) | 1.12 (0.92-1.33) |
| 8-21 | 28/5778 (0.5%) | 0.86 (0.53-1.18) |
| >21 | 3/405 (0.7%) | 1.72 (-0.25-3.69) |

##

## Supplementary Table S7. Maternal and pregnancy characteristics by first trimester paracetamol exposure in the matched cohort for the perinatal outcomes analysis

| **Characteristic** | **Paracetamol**  N = 39,917*^1^* | **Unexposed**  N = 217,368*^1^* | **aSMD before** | **aSMD after** |
| --- | --- | --- | --- | --- |
| **Calendar year of birth*^*^*** |  |  | 0.03 | 0.05 |
| 1998–2002 | 8,125 (20%) | 49,456 (23%) |  |  |
| 2003–2007 | 10,607 (27%) | 49,382 (23%) |  |  |
| 2008–2012 | 11,214 (28%) | 54,122 (25%) |  |  |
| 2013–2017 | 9,971 (25%) | 64,408 (30%) |  |  |
| **Maternal delivery age, years*^*^*** |  |  | 0.03 | 0.02 |
| <20 | 855 (2.1%) | 4,752 (2.2%) |  |  |
| 20–24 | 8,572 (21%) | 44,373 (20%) |  |  |
| 25–29 | 12,525 (31%) | 67,371 (31%) |  |  |
| 30–34 | 10,278 (26%) | 57,411 (26%) |  |  |
| 35–39 | 5,896 (15%) | 32,853 (15%) |  |  |
| 40–44 | 1,729 (4.3%) | 10,184 (4.7%) |  |  |
| ≥45 | 62 (0.2%) | 424 (0.2%) |  |  |
| **Maternal ethnic group (Bedouin)*^*^*** | 31,951 (80%) | 109,595 (50%) | 0.65 | 0 |
| **Maternal obesity** | 405 (1.0%) | 922 (0.4%) | 0.07 | 0.06 |
| **Maternal smoking during pregnancy*^*^*** | 161 (0.4%) | 762 (0.4%) | 0.01 | 0.02 |
| **Maternal diabetes*^*^*** | 301 (0.8%) | 629 (0.3%) | 0.06 | 0.02 |
| **Maternal comorbidity indicating Paracetamol Tx** | 5,430 (14%) | 19,431 (8.9%) | 0.15 | 0.1 |
| **Gravidity*^*^*** | 4 (2, 6) | 3 (2, 5) | 0.33 | 0.03 |
| **Gestational age, weeks*^*^*** | 39.6 (38.1, 40.3) | 39.3 (38.0, 40.1) | 0.04 | 0.01 |
| **Conception by assisted reproductive technology** | 161 (0.4%) | 727 (0.3%) | 0.01 | 0.04 |
| **Conception by insemination** | 39 (<0.1%) | 143 (<0.1%) | 0.01 | 0.03 |
| **Conception by IVF** | 137 (0.3%) | 627 (0.3%) | 0.01 | 0.04 |
| **Lack of prenatal care*^*^*** | 402 (1.0%) | 1,348 (0.6%) | 0.04 | 0.01 |
| **Folic acid** | 16,622 (42%) | 25,565 (12%) | 0.72 | 0.75 |
| **Sex of newborn (males)*^*^*** | 20,316 (51%) | 111,732 (51%) | 0.01 | 0.01 |
| **Major congenital malformations*^*^*** | 3,151 (7.9%) | 14,722 (6.8%) | 0.04 | 0 |
| *^1^*n (%); Median (Q1, Q3) | | | | |
| *^*^*Covariates included in adjusted models | | | | |
| aSMD, absolute Standardized Mean Difference; ATE, Average Treatment Effect; ESS, Effective Sample Size; IVF, In Vitro Fertilization; Tx, Treatment. | | | | |
| ESS for matching-adjusted model targeting the ATE were 19,945 exposed and 207,665 controls. No actual units were discarded during the proccess. | | | | |

## Supplementary Table S8. Maternal and pregnancy characteristics by first trimester paracetamol exposure in the matched cohort for the postnatal outcomes analysis

| **Characteristic** | **Paracetamol**  N = 39,917*^1^* | **Unexposed**  N = 217,368*^1^* | **aSMD before** | **aSMD after** |
| --- | --- | --- | --- | --- |
| **Calendar year of birth*^*^*** |  |  | 0.03 | 0 |
| 1998–2002 | 8,125 (20%) | 49,456 (23%) |  |  |
| 2003–2007 | 10,607 (27%) | 49,382 (23%) |  |  |
| 2008–2012 | 11,214 (28%) | 54,122 (25%) |  |  |
| 2013–2017 | 9,971 (25%) | 64,408 (30%) |  |  |
| **Maternal delivery age, years** |  |  | 0.03 | 0.09 |
| <20 | 855 (2.1%) | 4,752 (2.2%) |  |  |
| 20–24 | 8,572 (21%) | 44,373 (20%) |  |  |
| 25–29 | 12,525 (31%) | 67,371 (31%) |  |  |
| 30–34 | 10,278 (26%) | 57,411 (26%) |  |  |
| 35–39 | 5,896 (15%) | 32,853 (15%) |  |  |
| 40–44 | 1,729 (4.3%) | 10,184 (4.7%) |  |  |
| ≥45 | 62 (0.2%) | 424 (0.2%) |  |  |
| **Maternal ethnic group (Bedouin)*^*^*** | 31,951 (80%) | 109,595 (50%) | 0.65 | 0 |
| **Maternal obesity** | 405 (1.0%) | 922 (0.4%) | 0.07 | 0.04 |
| **Maternal smoking during pregnancy** | 161 (0.4%) | 762 (0.4%) | 0.01 | 0.01 |
| **Maternal diabetes** | 301 (0.8%) | 629 (0.3%) | 0.06 | 0.03 |
| **Maternal comorbidity indicating Paracetamol Tx*^*^*** | 5,430 (14%) | 19,431 (8.9%) | 0.15 | 0 |
| **Gravidity** | 4 (2, 6) | 3 (2, 5) | 0.33 | 0.17 |
| **Gestational age, weeks*^*^*** | 39.6 (38.1, 40.3) | 39.3 (38.0, 40.1) | 0.04 | 0.03 |
| **Conception by assisted reproductive technology** | 161 (0.4%) | 727 (0.3%) | 0.01 | 0.02 |
| **Conception by insemination** | 39 (<0.1%) | 143 (<0.1%) | 0.01 | 0.02 |
| **Conception by IVF** | 137 (0.3%) | 627 (0.3%) | 0.01 | 0.02 |
| **Lack of prenatal care** | 402 (1.0%) | 1,348 (0.6%) | 0.04 | 0.01 |
| **Folic acid** | 16,622 (42%) | 25,565 (12%) | 0.72 | 0.73 |
| **Sex of newborn (males)*^*^*** | 20,316 (51%) | 111,732 (51%) | 0.01 | 0 |
| *^1^*n (%); Median (Q1, Q3) | | | | |
| *^*^*Covariates included in adjusted models | | | | |
| aSMD, absolute Standardized Mean Difference; ATE, Average Treatment Effect; ESS, Effective Sample Size; IVF, In Vitro Fertilization; Tx, Treatment. | | | | |
| ESS for matching-adjusted model targeting the ATE were 19,909 exposed and 212,462 controls. No actual units were discarded during the proccess. | | | | |

## Supplementary Table S9. Peri- and post-natal outcomes for first trimester paracetamol exposure

| **First Trimester Exposure Ouctomes** | **Paracetamol**  N = 39,917 | **Unexposed**  N = 217,368 | **Unadjusted** | **Adjusted** | **Matched Adjusted** |
| --- | --- | --- | --- | --- | --- |
| **Perinatal Outcomes** | | | | | |
| **Preterm delivery** | 2,817 (7.1%) | 15,512 (7.1%) | 0.99 (0.95-1.03) | 0.97 (0.92-1.03) | 1.01 (0.94-1.08) |
| **Early preterm delivery** | 388 (1.0%) | 1,974 (0.9%) | 1.07 (0.96-1.19) | 1.04 (0.92-1.18) | 1.01 (0.88-1.16) |
| **APGAR < 7 at 1 min** | 1,593 (4.0%) | 8,714 (4.0%) | 1 (0.94-1.05) | 0.91 (0.86-0.97) | 0.88 (0.82-0.94) |
| **APGAR < 7 at 5 min** | 237 (0.6%) | 1,366 (0.6%) | 0.94 (0.82-1.08) | 0.83 (0.72-0.96) | 0.88 (0.73-1.07) |
| **APGAR < 7 at 1 OR 5 min** | 1,627 (4.1%) | 8,881 (4.1%) | 1 (0.95-1.05) | 0.92 (0.87-0.97) | 0.88 (0.82-0.94) |
| **Low birth weight** | 2,816 (7.1%) | 15,441 (7.1%) | 0.99 (0.95-1.03) | 0.99 (0.94-1.04) | 1.03 (0.97-1.1) |
| **Very low birth weight** | 326 (0.8%) | 1,678 (0.8%) | 1.06 (0.94-1.19) | 1.05 (0.93-1.18) | 1.03 (0.89-1.19) |
| **Perinatal death** | 543 (1.4%) | 2,660 (1.2%) | 1.11 (1.01-1.22) | 0.92 (0.83-1.01) | 0.96 (0.84-1.09) |
| **Postnatal Outcomes** | | | | | |
| **Markers for impaired renal functions** | 225 (0.6%) | 745 (0.3%) | 1.64 (1.42-1.91) | 1.08 (0.91-1.29) | 1.08 (0.87-1.33) |
| **Oligohydramnios** | 186 (0.5%) | 568 (0.3%) | 1.78 (1.51-2.1) | 1.11 (0.91-1.34) | 1.13 (0.89-1.45) |
| **Acute kidney injury** | 39 (<0.1%) | 177 (<0.1%) | 1.2 (0.85-1.7) | 1.07 (0.76-1.53) | 0.99 (0.66-1.47) |
| **Markers for premature ductus arteriosus closure** | 828 (2.1%) | 3,526 (1.6%) | 1.28 (1.19-1.38) | 1.11 (1.02-1.2) | 1.09 (0.99-1.21) |
| **Persistent fetal circulation** | 19 (<0.1%) | 81 (<0.1%) | 1.28 (0.78-2.11) | 1.28 (0.77-2.12) | 1.26 (0.72-2.21) |
| **Patent ductus arteriosus** | 773 (1.9%) | 3,337 (1.5%) | 1.26 (1.17-1.36) | 1.09 (1.01-1.19) | 1.07 (0.96-1.19) |
| **Primary pulmonary hypertension** | 43 (0.1%) | 162 (<0.1%) | 1.45 (1.03-2.02) | 1.19 (0.84-1.69) | 1.55 (0.98-2.45) |
| **Heart failure** | 10 (<0.1%) | 41 (<0.1%) | 1.33 (0.67-2.65) | 0.87 (0.43-1.74) | 0.92 (0.43-1.96) |

##

## Supplementary Table S10. Maternal and pregnancy characteristics by second trimester paracetamol exposure in the matched cohort for the perinatal outcomes analysis

| **Characteristic** | **Paracetamol**  N = 43,547*^1^* | **Unexposed**  N = 213,738*^1^* | **aSMD before** | **aSMD after** |
| --- | --- | --- | --- | --- |
| **Calendar year of birth*^*^*** |  |  | 0.06 | 0.04 |
| 1998–2002 | 9,162 (21%) | 48,419 (23%) |  |  |
| 2003–2007 | 11,725 (27%) | 48,264 (23%) |  |  |
| 2008–2012 | 12,132 (28%) | 53,204 (25%) |  |  |
| 2013–2017 | 10,528 (24%) | 63,851 (30%) |  |  |
| **Maternal delivery age, years*^*^*** |  |  | 0.02 | 0.04 |
| <20 | 911 (2.1%) | 4,696 (2.2%) |  |  |
| 20–24 | 9,260 (21%) | 43,685 (20%) |  |  |
| 25–29 | 13,689 (31%) | 66,207 (31%) |  |  |
| 30–34 | 11,158 (26%) | 56,531 (26%) |  |  |
| 35–39 | 6,508 (15%) | 32,241 (15%) |  |  |
| 40–44 | 1,940 (4.5%) | 9,973 (4.7%) |  |  |
| ≥45 | 81 (0.2%) | 405 (0.2%) |  |  |
| **Maternal ethnic group (Bedouin)*^*^*** | 34,396 (79%) | 107,150 (50%) | 0.63 | 0.01 |
| **Maternal obesity** | 387 (0.9%) | 940 (0.4%) | 0.06 | 0.04 |
| **Maternal smoking during pregnancy*^*^*** | 167 (0.4%) | 756 (0.4%) | 0 | 0.04 |
| **Maternal diabetes*^*^*** | 264 (0.6%) | 666 (0.3%) | 0.04 | 0.01 |
| **Maternal comorbidity indicating Paracetamol Tx** | 5,867 (13%) | 18,994 (8.9%) | 0.15 | 0.09 |
| **Gravidity*^*^*** | 4 (2, 6) | 3 (2, 5) | 0.33 | 0.05 |
| **Gestational age, weeks*^*^*** | 39.6 (38.0, 40.3) | 39.3 (38.0, 40.1) | 0.03 | 0.02 |
| **Conception by assisted reproductive technology** | 120 (0.3%) | 768 (0.4%) | 0.01 | 0.01 |
| **Conception by insemination** | 26 (<0.1%) | 156 (<0.1%) | 0.01 | 0 |
| **Conception by IVF** | 98 (0.2%) | 666 (0.3%) | 0.02 | 0.01 |
| **Lack of prenatal care*^*^*** | 459 (1.1%) | 1,291 (0.6%) | 0.05 | 0 |
| **Folic acid** | 17,313 (40%) | 24,874 (12%) | 0.68 | 0.71 |
| **Sex of newborn (males)*^*^*** | 22,232 (51%) | 109,816 (51%) | 0.01 | 0 |
| **Major congenital malformations*^*^*** | 3,435 (7.9%) | 14,438 (6.8%) | 0.04 | 0 |
| *^1^*n (%); Median (Q1, Q3) | | | | |
| *^*^*Covariates included in adjusted models | | | | |
| aSMD, absolute Standardized Mean Difference; ATE, Average Treatment Effect; ESS, Effective Sample Size; IVF, In Vitro Fertilization; Tx, Treatment. | | | | |
| ESS for matching-adjusted model targeting the ATE were 22,223 exposed and 202,459 controls. No actual units were discarded during the proccess. | | | | |

## Supplementary Table S11. Maternal and pregnancy characteristics by second trimester paracetamol exposure in the matched cohort for the postnatal outcomes analysis

| **Characteristic** | **Paracetamol**  N = 43,547*^1^* | **Unexposed**  N = 213,738*^1^* | **aSMD before** | **aSMD after** |
| --- | --- | --- | --- | --- |
| **Calendar year of birth*^*^*** |  |  | 0.06 | 0.01 |
| 1998–2002 | 9,162 (21%) | 48,419 (23%) |  |  |
| 2003–2007 | 11,725 (27%) | 48,264 (23%) |  |  |
| 2008–2012 | 12,132 (28%) | 53,204 (25%) |  |  |
| 2013–2017 | 10,528 (24%) | 63,851 (30%) |  |  |
| **Maternal delivery age, years** |  |  | 0.02 | 0.11 |
| <20 | 911 (2.1%) | 4,696 (2.2%) |  |  |
| 20–24 | 9,260 (21%) | 43,685 (20%) |  |  |
| 25–29 | 13,689 (31%) | 66,207 (31%) |  |  |
| 30–34 | 11,158 (26%) | 56,531 (26%) |  |  |
| 35–39 | 6,508 (15%) | 32,241 (15%) |  |  |
| 40–44 | 1,940 (4.5%) | 9,973 (4.7%) |  |  |
| ≥45 | 81 (0.2%) | 405 (0.2%) |  |  |
| **Maternal ethnic group (Bedouin)*^*^*** | 34,396 (79%) | 107,150 (50%) | 0.63 | 0 |
| **Maternal obesity** | 387 (0.9%) | 940 (0.4%) | 0.06 | 0.03 |
| **Maternal smoking during pregnancy** | 167 (0.4%) | 756 (0.4%) | 0 | 0.02 |
| **Maternal diabetes** | 264 (0.6%) | 666 (0.3%) | 0.04 | 0.01 |
| **Maternal comorbidity indicating Paracetamol Tx*^*^*** | 5,867 (13%) | 18,994 (8.9%) | 0.15 | 0 |
| **Gravidity** | 4 (2, 6) | 3 (2, 5) | 0.33 | 0.18 |
| **Gestational age, weeks*^*^*** | 39.6 (38.0, 40.3) | 39.3 (38.0, 40.1) | 0.03 | 0.02 |
| **Conception by assisted reproductive technology** | 120 (0.3%) | 768 (0.4%) | 0.01 | 0.02 |
| **Conception by insemination** | 26 (<0.1%) | 156 (<0.1%) | 0.01 | 0.01 |
| **Conception by IVF** | 98 (0.2%) | 666 (0.3%) | 0.02 | 0.02 |
| **Lack of prenatal care** | 459 (1.1%) | 1,291 (0.6%) | 0.05 | 0.01 |
| **Folic acid** | 17,313 (40%) | 24,874 (12%) | 0.68 | 0.7 |
| **Sex of newborn (males)*^*^*** | 22,232 (51%) | 109,816 (51%) | 0.01 | 0 |
| *^1^*n (%); Median (Q1, Q3) | | | | |
| *^*^*Covariates included in adjusted models | | | | |
| aSMD, absolute Standardized Mean Difference; ATE, Average Treatment Effect; ESS, Effective Sample Size; IVF, In Vitro Fertilization; Tx, Treatment. | | | | |
| ESS for matching-adjusted model targeting the ATE were 23,288 exposed and 208,335 controls. No actual units were discarded during the proccess. | | | | |

## Supplementary Table S12. Peri- and post-natal outcomes for second trimester paracetamol exposure

| **Second Trimester Exposure Ouctomes** | **Paracetamol** N = 43,547 | **Unexposed** N = 213,738 | **Unadjusted** | **Adjusted** | **Matched Adjusted** |
| --- | --- | --- | --- | --- | --- |
| **Perinatal Outcomes** | | | | | |
| **Preterm delivery** | 3,115 (7.2%) | 15,214 (7.1%) | 1 (0.97-1.04) | 0.97 (0.92-1.03) | 0.94 (0.86-1.03) |
| **Early preterm delivery** | 416 (1.0%) | 1,946 (0.9%) | 1.05 (0.94-1.17) | 0.97 (0.86-1.1) | 0.82 (0.7-0.97) |
| **APGAR < 7 at 1 min** | 1,795 (4.1%) | 8,512 (4.0%) | 1.04 (0.98-1.09) | 0.95 (0.9-1.01) | 0.94 (0.88-1) |
| **APGAR < 7 at 5 min** | 261 (0.6%) | 1,342 (0.6%) | 0.95 (0.84-1.09) | 0.84 (0.73-0.96) | 0.86 (0.73-1.03) |
| **APGAR < 7 at 1 OR 5 min** | 1,829 (4.2%) | 8,679 (4.1%) | 1.03 (0.98-1.09) | 0.95 (0.9-1.01) | 0.94 (0.88-1) |
| **Low birth weight** | 3,181 (7.3%) | 15,076 (7.1%) | 1.04 (1-1.08) | 1.02 (0.97-1.07) | 1.01 (0.95-1.07) |
| **Very low birth weight** | 360 (0.8%) | 1,644 (0.8%) | 1.07 (0.96-1.2) | 1 (0.89-1.12) | 0.93 (0.79-1.09) |
| **Perinatal death** | 615 (1.4%) | 2,588 (1.2%) | 1.17 (1.07-1.27) | 0.95 (0.86-1.05) | 1.04 (0.92-1.17) |
| **Postnatal Outcomes** | | | | | |
| **Markers for impaired renal functions** | 235 (0.5%) | 735 (0.3%) | 1.57 (1.35-1.82) | 1.04 (0.87-1.23) | 1.11 (0.9-1.36) |
| **Oligohydramnios** | 198 (0.5%) | 556 (0.3%) | 1.75 (1.49-2.06) | 1.08 (0.89-1.31) | 1.19 (0.94-1.51) |
| **Acute kidney injury** | 37 (<0.1%) | 179 (<0.1%) | 1.01 (0.71-1.45) | 0.9 (0.63-1.28) | 0.87 (0.56-1.34) |
| **Markers for premature ductus arteriosus closure** | 913 (2.1%) | 3,441 (1.6%) | 1.3 (1.21-1.4) | 1.12 (1.04-1.21) | 1.05 (0.96-1.16) |
| **Persistent fetal circulation** | 17 (<0.1%) | 83 (<0.1%) | 1.01 (0.6-1.69) | 1.03 (0.62-1.74) | 0.89 (0.51-1.55) |
| **Patent ductus arteriosus** | 859 (2.0%) | 3,251 (1.5%) | 1.3 (1.2-1.4) | 1.12 (1.04-1.21) | 1.06 (0.96-1.17) |
| **Primary pulmonary hypertension** | 47 (0.1%) | 158 (<0.1%) | 1.46 (1.05-2.02) | 1.19 (0.85-1.67) | 0.96 (0.67-1.38) |
| **Heart failure** | 17 (<0.1%) | 34 (<0.1%) | 2.45 (1.37-4.39) | 1.63 (0.89-2.97) | 1.47 (0.78-2.75) |

## Supplementary Table S13. Distribution of Indications for Analgesic Use, Grouped by Category, in Exposed vs Unexposed Pregnancies

| **Characteristic** | **Paracetamol** N = 41,032*^1^* | **Unexposed** N = 224,111*^1^* | **p-value*^2^*** |
| --- | --- | --- | --- |
| **Musculoskeletal** | 7 (<0.1%) | 2 (<0.1%) | <0.001 |
| **Pain / Inflammatory** | 12 (<0.1%) | 115 (<0.1%) | 0.060 |
| **Injury / Fractures** | 897 (2.2%) | 3,144 (1.4%) | <0.001 |
| **Pregnancy Related Indications** | 4,094 (10.0%) | 15,156 (6.8%) | <0.001 |
| **Fever / Infection** | 1,447 (3.5%) | 4,217 (1.9%) | <0.001 |
| *^1^*n (%) | | | |
| *^2^*Fisher's exact test; Pearson's Chi-squared test | | | |

Paracetamol indications were identified using ICD-9 diagnostic codes recorded during the first trimester of pregnancy, corresponding to clinical conditions for which paracetamol is commonly used as an analgesic or antipyretic. These included febrile or infectious illnesses (e.g., general fever 780.6, 780.60; respiratory and ENT infections 460–466, 381*, 462–463; urinary tract infections 599.0, 590*; other infectious or inflammatory conditions 041*, 079*, 682*, 614*, 615*), pain-related conditions (e.g., headache 346, abdominal or pelvic pain 789, 623–625), musculoskeletal and joint disorders (e.g., back pain, myalgia, arthritis 714–716, 718–719, 724, 729), injuries or fractures (800–829, 840–848, 920–924), and pregnancy-related diagnoses potentially associated with analgesic use, such as threatened abortion (632). Women could have more than one indication, and all relevant first-trimester diagnoses were combined into a single composite indicator for paracetamol indication (yes/no).
